# Proteogenomic characterization of difficult-to-treat breast cancer with tumor cells enriched through laser microdissection

**DOI:** 10.1186/s13058-024-01835-4

**Published:** 2024-05-14

**Authors:** Praveen-Kumar Raj-Kumar, Xiaoying Lin, Tao Liu, Lori A. Sturtz, Marina A. Gritsenko, Vladislav A. Petyuk, Tyler J. Sagendorf, Brenda Deyarmin, Jianfang Liu, Anupama Praveen-Kumar, Guisong Wang, Jason E. McDermott, Anil K. Shukla, Ronald J. Moore, Matthew E. Monroe, Bobbie-Jo M. Webb-Robertson, Jeffrey A. Hooke, Leigh Fantacone-Campbell, Brad Mostoller, Leonid Kvecher, Jennifer Kane, Jennifer Melley, Stella Somiari, Patrick Soon-Shiong, Richard D. Smith, Richard J. Mural, Karin D. Rodland, Craig D. Shriver, Albert J. Kovatich, Hai Hu

**Affiliations:** 1 Chan Soon-Shiong Institute of Molecular Medicine at Windber, Windber, PA USA; 2 Murtha Cancer Center Research Program, Department of Surgery, Uniformed Services University of the Health Sciences, Bethesda, MD USA; 3 Pacific Northwest National Laboratory, Richland, WA USA; 4 The Henry M. Jackson Foundation for the Advancement of Military Medicine, Inc, Bethesda, MD USA; 5 NantWorks, Culver City, CA USA; 6 Department of Surgery, Walter Reed National Military Medical Center, Bethesda, MD USA

**Keywords:** Breast cancer, Laser microdissection, Proteogenomics, Phosphoproteomics

## Abstract

**Background:**

Breast cancer (BC) is the most commonly diagnosed cancer and the leading cause of cancer death among women globally. Despite advances, there is considerable variation in clinical outcomes for patients with non-luminal A tumors, classified as difficult-to-treat breast cancers (DTBC). This study aims to delineate the proteogenomic landscape of DTBC tumors compared to luminal A (LumA) tumors.

**Methods:**

We retrospectively collected a total of 117 untreated primary breast tumor specimens, focusing on DTBC subtypes. Breast tumors were processed by laser microdissection (LMD) to enrich tumor cells. DNA, RNA, and protein were simultaneously extracted from each tumor preparation, followed by whole genome sequencing, paired-end RNA sequencing, global proteomics and phosphoproteomics. Differential feature analysis, pathway analysis and survival analysis were performed to better understand DTBC and investigate biomarkers.

**Results:**

We observed distinct variations in gene mutations, structural variations, and chromosomal alterations between DTBC and LumA breast tumors. DTBC tumors predominantly had more mutations in *TP53*, *PLXNB3*, Zinc finger genes, and fewer mutations in *SDC2*, *CDH1*, *PIK3CA*, *SVIL*, and *PTEN*. Notably, Cytoband 1q21, which contains numerous cell proliferation-related genes, was significantly amplified in the DTBC tumors. LMD successfully minimized stromal components and increased RNA–protein concordance, as evidenced by stromal score comparisons and proteomic analysis. Distinct DTBC and LumA-enriched clusters were observed by proteomic and phosphoproteomic clustering analysis, some with survival differences. Phosphoproteomics identified two distinct phosphoproteomic profiles for high relapse-risk and low relapse-risk basal-like tumors, involving several genes known to be associated with breast cancer oncogenesis and progression, including *KIAA1522*, *DCK*, *FOXO3*, *MYO9B*, *ARID1A*, *EPRS*, *ZC3HAV1*, and *RBM14*. Lastly, an integrated pathway analysis of multi-omics data highlighted a robust enrichment of proliferation pathways in DTBC tumors.

**Conclusions:**

This study provides an integrated proteogenomic characterization of DTBC vs LumA with tumor cells enriched through laser microdissection. We identified many common features of DTBC tumors and the phosphopeptides that could serve as potential biomarkers for high/low relapse-risk basal-like BC and possibly guide treatment selections.

**Supplementary Information:**

The online version contains supplementary material available at 10.1186/s13058-024-01835-4.

## Introduction

Breast cancer (BC) is the most commonly diagnosed cancer and the leading cause of cancer death in women worldwide [1]. BC is classified into four widely-accepted intrinsic subtypes based on PAM (Prediction Analysis of Microarray) 50 gene expression profiles: basal-like (Basal), Her2-enriched (Her2), Luminal B (LumB) and Luminal A (LumA) [2]. BC can also be subtyped based on 4 immunohistochemistry (IHC) markers (estrogen receptor (ER), progesterone receptor (PR), human epidermal growth factor receptor 2 (HER2) and Ki67 [3–5]) as triple-negative (TN), HER2 + , luminal B1 (LB1), luminal B2 (LB2) and luminal A (LA). PAM50-based intrinsic subtypes are commonly used in gene expression studies whereas IHC-based subtypes are often used for guiding clinical interventions [4, 6, 7]. There is up to 35% discordance between IHC subtypes and intrinsic subtypes [8]. We recently published a method called PCA-PAM50 which is a refined approach of the PAM50 classifier and improves concordance of IHC and intrinsic subtypes by up to 9% [9]. This subtype discordance adds further complexity to understanding variations in clinical outcomes of patients with DTBC and luminal A breast tumors. Proteogenomic profiling of these tumors would further enhance our molecular understanding of these tumors while also providing additional insight into the causes of the observed outcome differences.

Recent multi-omics studies of human BC have identified many potential therapeutic biomarkers for each of these BC subtypes [10–13]. However, there is still considerable variation in the clinical outcomes of patients with DTBC tumors [14–17], most likely due to the extensive molecular heterogeneity of the disease. On the other hand, LumA patients have better outcomes because this tumor subtype is typically slower-growing and responsive to hormone therapy [18]. Hormone therapy, such as tamoxifen or an aromatase inhibitor, works by blocking the effects of estrogen on the cancer cells, thus slowing or stopping the growth of the tumor [19].

A common feature of DTBC tumors is that they do not respond well to existing therapies. For example, despite the clinical benefits of HER2-targeted therapies, many HER2 + tumors develop resistance to targeted therapy [20] and will eventually develop progressive disease. LumB BC is defined by aggressive clinical behavior and has a prognosis similar to that of other DTBC [21]. Likewise, there are limited targeted therapies for triple negative primary tumors which have elevated immune infiltration and DNA repair activities [22], and the majority of these patients experience relapse within the first 5 years of diagnosis [14, 23–25].

Here, we strive to understand the proteogenomic characteristics of intrinsically-defined DTBC tumors in reference to LumA tumors. We used the IHC subtype to enrich our cohort with non-LA tumors during sample selection. Proteogenomics involves the integrative analysis of DNA sequencing, RNA sequencing, and mass spectrometry (MS)-based proteomics and phosphoproteomics to provide a comprehensive picture of the impact of genotype on phenotype [26, 27].

One major challenge in BC research is the heterogeneous nature of breast tumor specimens where a varying percentage of surrounding non-cancer tissues may interfere with the study of cancer cells [28]. Historically, breast cancer studies were performed using samples from bulk processing, which included both tumor and stroma. However, the proportions of tumor cells in the samples vary considerably depending on the tumor purity. For example, the tumor purity of The Cancer Genome Atlas (TCGA)-BC study [29] bulk-processed samples ranged from 60 to 95%. To better capture the proteogenomic landscape of breast cancer, we analyzed relatively pure populations of tumor cells from breast tumor specimens using LMD, a method that allows direct microscopic visualization of the specimen and collection of specific cell types [28, 30]. Furthermore, we also simultaneously extracted all three molecules (DNA, RNA, and protein) from the same LMD-collected samples, thus enabling more precise integrative analyses. Another important advantage of this study is that 34 tumors in our cohort were also previously used for the bulk processing-based TCGA-BC study [29] which enabled a side-by-side comparison of LMD and bulk processing. To our knowledge, this is the first proteogenomic study of BC using LMD-processed tumors and simultaneous genomic, transcriptomic, and proteomic analysis performed on the same samples.

## Materials and methods

### Human subjects and consent to participate

Data collection was conducted in accordance with a research protocol entitled “Tissue and Blood Library Establishment for Molecular, Biochemical and Histologic Study of Breast Disease”, approved by the IRB of the Walter Reed National Military Medical Center (IRBNet #20,704) for the Clinical Breast Care Project (CBCP) [31]. We followed the proper guidelines to obtain publicly available TCGA data.

### Sample collection

Fresh breast tissue specimens were collected from patients following excisional biopsy from 2001 to 2010. After undergoing gross pathology assessment, breast tissue specimens were embedded in Optimal Cutting Temperature (OCT) compound, quick-frozen, and stored at − 180 °C in liquid nitrogen freezers at the CBCP Biobank in Windber, PA.

### IHC subtypes

IHC subtyping was used to enrich the cohort with non-LA tumors. The IHC subtypes for 117 primary breast cancer tissue samples were determined using the IHC assays for ER, PR, HER2, and Ki67 in a centralized CLIA-certified laboratory following standardized protocols as defined previously [9]. The study cohort included 30 triple negative (TN; ER−/PR−/HER2−), 16 HER2+(ER−/PR−/HER2+), 39 Luminal B1 (LB1; ER+/HER2−/Ki67+), 17 Luminal B2 (LB2; ER+/HER2+) and 15 Luminal A (LA; ER+/HER2−/Ki67−) subtypes.

### Laser microdissection and molecular extraction

Optimal Cutting Temperature compound (OCT)-embedded breast tumors were processed by laser microdissection (LMD) to collect and enrich for tumor cells. OCT-embedded specimens were sectioned at 8 µm inside a temperature-controlled cryostat (Leica Microsystems, Buffalo Grove, IL) and mounted on polyethylene-naphthalate (PEN) membrane slides (W. Nuhsbaum Inc, McHenry, IL). Scout slides were created by mounting every 10th section on microscopic plus slides and staining with hematoxylin and eosin (H&E), and regions of interest (ROI) for LMD were marked by a pathologist (JAH). Next, PEN membrane slides were stained with cresyl violet staining solution (Ambion/Applied Biosystems, Grand Island, NY), and LMD performed according to the marked ROI using the Leica ASLMD system. Following LMD, the collected sample was incubated for 10 min at 37 °C in an air incubator. After incubation, the sample was vortexed briefly, a quick spin performed, and the sample pipetted up and down several times before transferring the lysate to a DNA column. DNA, RNA, and protein were then simultaneously extracted from each tumor specimen using the Illustra triplePrep kit (Cytiva, Marlborough, MA) following the manufacturer’s protocol. The optional DNase treatment of the RNA was performed. Protein pellets were washed 2–3 times with 1 mL of nuclease-free water and then re-suspended in 100 µl of 8M urea in 100 mM ammonium bicarbonate, pH 7.8. Following isolation with the triplePrep kit, the tumor DNA samples were further cleaned up using the Genomic DNA Clean & Concentrator-10 kit (Zymo Research Corporation, Irvine, CA) to remove protein contaminants. The concentrations of the DNA, RNA and protein samples were measured using the Qubit fluorometer (Thermo Fisher Scientific Inc., Waltham, MA), and the integrity of the RNA samples was determined using the Bioanalyzer (Agilent Technologies, Inc., Santa Clara, CA). Germline blood DNA (“normal”) from clots was extracted from BD Vacutainer 10 mL serum collection tubes (Becton, Dickinson and Company, Franklin Lakes, NJ) using the Gentra Puregene Blood Kit (Qiagen Sciences, Germantown, MD), and the concentrations measured using the Qubit fluorometer. Tumor DNA and germline DNA samples were normalized to final concentrations of 5 ng/µl in a total volume of 100 µl and 10 ng/µl in a total volume of 50 µl, respectively, for whole genome sequencing (WGS). RNA samples were diluted to 50ng/µl for total RNA sequencing (RNA-Seq).

### RNA-Seq data and analysis

RNA-Seq libraries were prepared for 117 tumor samples using the KAPA Stranded RNA-Seq Kit with RiboErase (Kapa Biosystems, Wilmington, MA). Paired-end total RNA-Seq with 150 nt reads and a 200 nt insert was performed using the Illumina HiSeq platform to produce a minimum of 100 million sequencing reads, and data supplied as a BAM file. We used the BEDTools [32] bamtofastq utility to convert the BAM file into fastq files. FastQC [33] was used to check the quality of the reads, and the data preprocessed using PRINSEQ [34] version 0.20.427 to trim low-quality bases (≤ 20) and poly A/T/N tails; the minimum length retained was 35 nt. STAR [35] version 2.7.0f was used for splice alignment to reference genome hg19 from ENSEMBL release 75 [36]. The featureCounts [37] and HTSeq [38] was used to quantify gene expression with the guidance of the gene annotation file GTF from ENSEMBL release 75. Gene expression was upper quartile normalized and log transformed. Genes with a mean expression of at least 10 reads were considered for downstream analysis. This identified a total of 26,236 genes which included 16,690 protein coding genes and 9546 non-coding genes (pseudogene, antisense, lncRNA, etc.). Differential gene expression was performed using DESeq2 [39] and Limma-Voom [40]. The intrinsic subtypes were derived using the PCA-PAM50 [9] method, which is a refined model of the PAM50 method [2]. Scripts were written in Perl and R programming languages.

In order to compare the 34 overlapping cases between TCGA [29] and our cohort (bulk vs. LMD, respectively), the RNA-Seq raw count data from TCGA was obtained from the harmonized database via the Genomic Data Commons (GDC) data portal (dbGaP Study Accession phs000178 for TCGA-BRCA) using the TCGAbiolinks [41] Bioconductor package. This gave us the unique opportunity to perform a side-by-side comparison of stromal, immune and microenvironment scores for those cases. The htseq count data were upper quartile normalized and log transformed. The 3 normal-like PAM50 subtype cases among these 34 were replaced with the next best subtype using the PAM50 classifier scores. The xCell [42] algorithm was used for generating the stromal, immune, and microenvironment scores. The recently published tool ComBat-seq [43] was used to compare differential gene expression between LMD and TCGA. ComBat-seq uses untransformed, raw count matrix combined with a known batch variable as input and outputs negative binomial regression adjusted data. The combined LumA cases of TCGA and LMD were then used to perform differential expression analysis adjusted by covariate histology.

### WGS data and analysis

Libraries for WGS were prepared using the KAPA Hyper prep kit (Kapa Biosystems, Wilmington, MA) for 99 tumors and their respective matching germline blood sample (“normal”). WGS with 2 × 150 sequencing and a 300 nt insert was performed using the Illumina HiSeq instrument to provide minimum coverage of 50 × for tumor samples and 25 × for matched normal germline samples. Mapping was done using BWA-MEM [44] against the hg19 human reference sequence. The other 18 (117–99) tumors from this cohort did not undergo WGS either due to lack of a matching germline blood sample or a quality failure.

#### Somatic and germline mutation analysis

The WGS BAM files were processed by marking duplicates and re-ordering with Picard tools (version 2.9.0) to produce analysis-ready BAMs for tumor and matched-normal pairs of each of the 99 cases. A quality check was performed using genotype match analyses following recommendations from Conpair [45] and BAM-matcher [46]. Single nucleotide variants (SNVs) and INDELs (insertions/deletions) were called using Strelka2 [47] and Manta [48]. Somatic mutations were annotated with ANNOVAR [49] and Ensembl VEP [50] and then converted to a MAF file using vcf2maf tool (https://github.com/mskcc/vcf2maf). The annotated variants were filtered for protein-altering events including non-synonymous SNVs, frameshift INDELs, non-frameshift INDELs, missense mutations and stop gains. Maftools [51] was used for generating oncoplots. MuSiC2 [52] was used to call the significantly mutated genes (SMG) above the background mutation rate. A false discovery rate (FDR) of 5% was used as the cutoff to identify SMG. Firth logistical regression [53] was used to find the differentially mutated genes (DMG) between DTBC and LumA tumors (*p* < 0.1). The genes commonly identified between SMG and DMG formed the significantly differentially mutated genes. Finally, the tumor mutational burden (TMB) was measured as the number of non-synonymous somatic mutations.

#### Somatic copy number alteration analysis

Sequenza 3.0.0 (26) was used to identify somatic copy number alterations (SCNAs) using normal samples as a reference. The WGS-derived analysis-ready BAMs were used with Sequenza to produce copy number segments, allele-specific copy numbers, tumor purity and tumor ploidy for each patient. Default settings were used following the recommendations of the manual. Briefly, copy number profiles were inferred by using the relative number of reads mapped to a given genomic position in tumor versus normal (depth ratio). The depth ratios were normalized using the mean ratio of each GC window and the respective GC content. GISTIC2.0 (27) (version 2.0.23) was used to identify significantly amplified and deleted regions in the cohort. Output segmentations from Sequenza were used as the input for GISTIC2.0 following the recommendations in the manual. GISTIC parameters were set to default values except the maxseg parameter was set to 8500 in order to accommodate the number of segments in some Basal cases. GISTIC2.0 generated arm level and focal level SCNAs for the cohort with the G-Score and FDR Q value indicating the significance and strength of the identified SCNAs, and relative SCNA was calculated as 2 ^ log2(absolute somatic copy number) – 1.

To identify candidate SCNA driver genes, we selected all of the genes associated with chromosomal focal level changes as identified by GISTIC2.0 q-value less than 0.25. For these genes, Pearson correlations were calculated between copy number values and their RNA levels across the cohort. An absolute correlation coefficient greater than 0.3 was chosen as the cutoff to select candidate genes.

### Global proteomics and phosphoproteomics analysis

#### Tryptic digestion of proteins

Approximately 400 μg of proteins from 100 μL of each sample were diluted and re-suspended using 300 µL of lysis buffer (8 M urea, 100 mM NH_4_HCO_3_, pH 8.0, 10 mM NaF, phosphatase inhibitor cocktail 2, phosphatase inhibitor cocktail 3, 20 μM PUGNAc). Lysates were pre-cleared by centrifugation at 16,500 g for 5 min at 4 °C and protein concentrations were determined by BCA assay (Pierce). Proteins were reduced with 5 mM dithiothreitol for 1 h at 37 °C and subsequently alkylated with 10 mM iodoacetamide for 1 h at 25 °C in the dark. Samples were diluted 1:2 with 100 mM NH_4_HCO_3_, 1 mM CaCl_2_ and digested with sequencing-grade modified trypsin (Promega) at 1:50 enzyme‐to‐substrate ratio. After 4 h of digestion at 37 °C, samples were diluted 1:4 with the same buffers and another aliquot of the same amount of trypsin was added to the samples and further incubated at 25 °C overnight (16 h). The digested samples were then acidified with 10% trifluoroacetic acid to ~ pH 3. Tryptic peptides were desalted on strong cation exchange SPE (Supelco) and reversed-phase C18 SPE columns (Supelco) and dried using a Speed-Vac.

#### TMT-6 labeling

The desalted peptides from each sample were labeled with 6-plex Tandem Mass Tag (TMT) reagents according to the manufacturer’s instructions (ThermoScientific). Peptides (100 µg) from each of the samples were dissolved in 30 μL of 500 mM triethylammonium bicarbonate, pH 8.5, and mixed with one unit of TMT reagent that was dissolved freshly in 70 μL of anhydrous acetonitrile. Channel 131 was used for labeling the pooled internal reference sample (pooled from all tumor samples with equal contribution) throughout the sample analysis. After a 1 h incubation at RT, 8 µL of 5% hydroxylamine was added and incubated for 15 min at RT to quench the reaction. Peptides labeled by different TMT reagents were then mixed, dried down to ~ 250 μL using a Speed-Vac, and desalted on C18 SPE columns.

#### Peptide fractionation by basic reversed-phase liquid chromatography

Approximately 400 μg of 6-plex TMT-labeled sample was separated on a Waters reversed-phase XBridge C18 column (250 mm × 4.6 mm column containing 5-μm particles, and a 4.6 mm × 20 mm guard column) using an Agilent 1200 HPLC System. After sample loading, the C18 column was washed for 35 min with solvent A (10 mM ammonium formate, pH 7.5), before applying a 112-min LC gradient with solvent B (10 mM ammonium formate, pH 7.5, 90% acetonitrile). The LC gradient began with a linear increase of solvent A to 10% B in 6 min, then linearly increased to 30% B in 86 min, 10 min to 42.5% B, 5 min to 55% B, and 5 min to 100% B. The gradient then resolved to 100% solvent A in 1 min and kept at 100% A for 30 min. The flow rate was 0.5 mL/min. A total of 96 fractions were collected from 48 to 164 min of the LC gradient into a 96-well plate (1.2 mL per fraction). Fractions 1–75 were concatenated into 12 fractions by combining the fractions that are 13 fractions apart; fractions 76–96 were pooled as a 13th fraction. For proteome analysis, 5% of each of the 12 concatenated fractions was dried and re-suspended in 2% acetonitrile, 0.1% formic acid to a peptide concentration of 0.1 μg/μL for LC–MS/MS analysis. The remainder of the 12 concatenated fractions (95%) were further concatenated into six fractions by combining two concatenated fractions (i.e., combining concatenated fractions #1 and #7; #2 and #8; and so on), dried, and subjected to immobilized metal affinity chromatography (IMAC) for phosphopeptide enrichment. The 13th fraction was not split and combined further, like the other fractions, and it was subjected to IMAC enrichment directly; the resulting eluant was analyzed as the 7th phosphoproteome fraction, and the IMAC flow-through was analyzed as the 13th global proteome fraction.

#### Phosphopeptide enrichment using IMAC

Fe^3+^-NTA-agarose beads were freshly prepared using Ni–NTA magnetic agarose beads (QIAGEN) for phosphopeptide enrichment. For each of the six fractions from the same TMT-6 plex, peptides were reconstituted in 135 μL IMAC binding/wash buffer (80% acetonitrile, 0.1% TFA) and incubated with end-over-end rotation with 35 μL of the 50% bead suspension for 30 min at RT. After incubation, the beads were washed four times each with 150 μL of wash buffer. Phosphopeptides were eluted from the beads using 50 μL of elution buffer (1:1 acetonitrile: 5% ammonia water in 5 mM pH 8 phosphate buffer, pH ~ 10), and acidified immediately to pH 3.5–4 with 10% TFA. Samples were dried using a Speed-Vac and later reconstituted with 20 μL of 3% acetonitrile, 0.1% formic acid for LC–MS/MS analysis.

#### LC–MS/MS analysis

The global proteome and phosphoproteome fractions were separated using a Waters nano-Acquity dual pumping UPLC system (Milford, MA) custom configured for on-line trapping of a 10-µL injection at 3 µL/min with reverse direction elution onto the analytical column at 300 nL/min. Columns were packed in-house using 360-µm o.d. fused silica (Polymicro Technologies Inc., Phoenix, AZ) with 5-mm sol–gel frits for media retention [54] and contained Jupiter C18 media (Phenomenex, Torrence, CA) in 5-µm particle size for the trapping column (150 µm i.d. × 4 cm long) and 3-µm particle size for the analytical column (75 µm i.d. × 70 cm long). Mobile phases consisted of (A) 0.1% formic acid in water and (B) 0.1% formic acid in acetonitrile with the following gradient profile (min, %B): 0, 1; 2, 8; 20, 12; 75, 30; 97, 45; 100, 95; 110, 95; 115, 1; 150, 1.

MS analysis was performed using a Q-Exactive Plus mass spectrometer (Thermo Scientific, San Jose, CA) outfitted with a homemade nano-electrospray ionization interface. Electrospray emitters were homemade using 150 µm o.d. × 20 µm i.d. chemically etched fused silica [55]. The heated capillary temperature and spray voltage were 325 °C and 2.3 kV, respectively. Data were collected for 100 min following a 15 min delay from sample injection. Orbitrap precursor spectra (AGC 1 × 10^6^) were collected from 400 to 2000 m/z at a resolution of 35,000 with the top-ten data-dependent Orbitrap HCD MS/MS spectra at a resolution of 17,500 (AGC 1 × 10^5^) and max ion time of 100 ms. Masses selected for MS/MS were isolated at a width of 2.0 m/z and fragmented using a normalized collision energy of 30% and a dynamic exclusion time of 30 s.

#### Proteomics data processing

The Thermo RAW files were converted to mzML format using the msConvert tool in ProteoWizard [56]. These files were used to search against the reference proteome hg19 from Ensembl release 75. The partially tryptic search used a ± 10 ppm parent ion tolerance, allowed for isotopic error in precursor ion selection, and searched a decoy database composed of the forward and reverse protein sequences. MS-GF + [57, 58]considered static carbamidomethylation (+ 57.0215 Da) on Cys residues, TMT modifications (+ 229.1629 Da) on peptide N termini and Lys residues, and dynamic oxidation (+ 15.9949 Da) on Met residues for searching the global proteome data. Peptide identification stringency was set to a maximum FDR of 1% at the peptide level using PepQValue < 0.005 and parent ion mass deviation < 8 ppm criteria. A minimum of 6 unique peptides per 1000 amino acids of protein length was required for achieving 1% at the protein level within the full dataset. Inference of the parsimonious protein set resulted in the identification of a total of 8,019 common protein groups among the 112 samples. Phosphopeptides were identified from the phosphoproteomics data files as described above (e.g., peptide level FDR < 1%), with an additional dynamic phosphorylation (+ 79.9663 Da) on Ser, Thr, or Tyr residues. The phosphoproteome data were further processed by the Ascore algorithm^70^ for phosphorylation site localization, and the top-scoring sequences were reported. Prioritized protein inference (proteins that passed inference in global) was kept and shared peptides were dropped.

The intensities of all six TMT reporter ions were extracted using MASIC software^44^. Next, PSMs were linked to the extracted reporter ion intensities by scan number. The reporter ion intensities from different scans and different fractions corresponding to the same protein or phosphopeptide were summed. Relative protein or phosphopeptide abundance was calculated as the ratio of abundance in a given sample to the reference abundance. The pooled reference sample was labeled with TMT 131 reagent, allowing comparison of relative protein or phosphopeptide abundances across different TMT-6 plexes. The relative abundances were log2 transformed and zero-centered for each protein and phosphopeptide to obtain final, relative abundance values. Sample quality control (QC) of the quantified proteins was performed using a density plot which demonstrated that all samples conformed to an expected unimodal distribution. Principal component analyses (PCA) were performed to confirm that there were no sequencing batch effects after normalization.

#### Proteome and phosphoproteome clusters

Robust clusters were derived with consistently detected proteins and phosphopeptides in all tumors for proteomics and phosphoproteomics, respectively. In the case of phosphoproteomic data, there were 331 consistently detected phosphopeptides associated with 245 unique genes and 245 unique proteins. We aimed to select a unique phosphopeptide for each gene; therefore, from the many phosphopeptides for a gene, we selected the one with the highest variation based on the standard deviation metric. For proteomic data, there were 1461 consistently detected proteins, corresponding to 1461 unique proteins and 1457 unique genes. We sought to select unique gene proteins; thus, from the many proteins for a gene, we chose the one with the highest variation based on the standard deviation metric. These values were median centralized and used for clustering. Consensus clustering was performed using the ConsensusClusterPlus [59] R Bioconductor package. The features were transformed into 1000 bootstrap sample data sets with a probability of 0.8 for selecting any sample and any protein. The bootstrap data sets were clustered using k-means clustering with up to 6 clusters. Based on both visual inspection of the consensus matrix and the silhouette plots for identifying better coherence, the clusters were selected.

#### Mertins et al. 2016 dataset

The proteome and phosphoproteome dataset from this study was obtained from the supplemental data of Mertins et al. 2016, as well as through personal communication with the corresponding author of the study. To compare the ESTIMATE scores of the proteome clusters from our data with those in the Mertins et al. 2016 paper, we communicated with the corresponding author and obtained the necessary data.

#### Protein-mRNA correlation

Gene-wise Pearson correlation coefficients were calculated for each mRNA and protein pair, including mRNA from RNA-Seq and protein from global proteomics, across the cohort. Sample-wise Pearson correlation coefficients were calculated for each sample’s mRNA and protein features. To derive the correlation for the Mertins et al. 2016 study, we obtained their protein data from the supplemental files, and the relevant RNA-Seq data was taken from the TCGA-BRCA dataset as previously mentioned. Correlation coefficients and FDR adjusted p-values were calculated in R.

#### Treatment data analysis

Treatment data, when available, was obtained for our entire cohort. The drug names were cleaned and classified into four categories: chemotherapy, hormone therapy, HER2-targeted therapy, and radiation therapy. The drug names and classes are included in Table S1A. For our analysis of the data presented in Fig. 4C, the ‘Unknown’ category was converted to 'NA' for each treatment type and treated as standard missing data. After this conversion, certain treatment types, such as chemotherapy and radiotherapy, were found to have insufficient levels; specifically, they only possessed one level, 'Yes', and lacked a 'No' level. For Cox proportional hazards regression models (coxph) or the Fisher exact test to be valid, at least two levels are required. Therefore, we cannot include chemotherapy and radiotherapy in our analysis. Most of the basal cases are TN per IHC and are expected to receive only chemotherapy and radiotherapy, thus using hormone therapy and/or HER2-targeted therapy as factors is invalid for assessing their influence on survival differences in basal cases. Furthermore, the Fisher exact test result for hormone therapy and HER2-targeted therapy between the high and low relapse-risk groups produced a *p*-value of 1, indicating no significant difference between the treatments given to these two groups.

### Statistical analysis

All statistical analyses were carried out in R (version 4.1.0). PCA analysis was performed using PCAtools [60] in the Bioconductor package. The unsupervised hierarchical clustering analysis was performed with the heatmap3 [61] and ComplexHeatmap [62] Bioconductor packages. Kaplan–Meier plots and log-rank tests for statistical significance were executed using the survival package in R. Wilcoxon rank sum test was used for statistical significance (*p* < 0.05) with numeric features, unless indicated otherwise. Fisher exact test was used for statistical significance (*p* < 0.05) with categorical features, unless indicated otherwise. The clinical endpoints of overall survival (OS) and progression-free Interval (PFI) were derived as previously described [63].

### Pathway analysis

#### Gene set enrichment analysis (GSEA)

GSEA [64] was used on transcripts or global protein expression data for Cancer Hallmark Pathway analysis (database h.all.v2023.1.Hs.symbols.gmt [65]). The differentially expressed gene or protein list and the corresponding statistic t were used as input in the GSEA Preranked tool in GSEA software, where t values were used for ranking the genes. FDR < 0.05 was applied to get significant pathways.

#### Multi-omics gene set analysis

The multi-omics gene set analysis (MOGSA) [66] software package (version 1.22.1) in R was used to perform multivariate single sample gene-set analysis. Briefly, we calculated the integrated single sample gene-set scores (GSS) of MSigDB hallmark gene-set pathways from transcriptomic and global proteomic data using the first 5 principal components. To identify pathways enriched in the DTBC and LumA groups, we first selected pathways in which individual sample GSS *p*-values were < 0.01 in at least 50% of all samples. From these pathways, we used Generalized Linear Model (GLM) to estimate the difference in sample GSS values between the two groups, and a *p*-value < 0.01 was used to identify significantly different pathways. The direction up/down in a group was determined by the sign of the t-value from the GLM model.

#### Ingenuity pathway analysis

Regulatory network analysis to predict functional regulatory networks was performed using Ingenuity Pathway Analysis (IPA) software [67]. Recommended log fold-change (FC) values and FDR-adjusted* p*-values of significantly differentially expressed genes or proteins served as the input for IPA.

## Results

### Proteogenomic profiling of LMD breast tumors reveals reduced stromal and immune contributions in LumA LMD samples

**Fig. 1:**
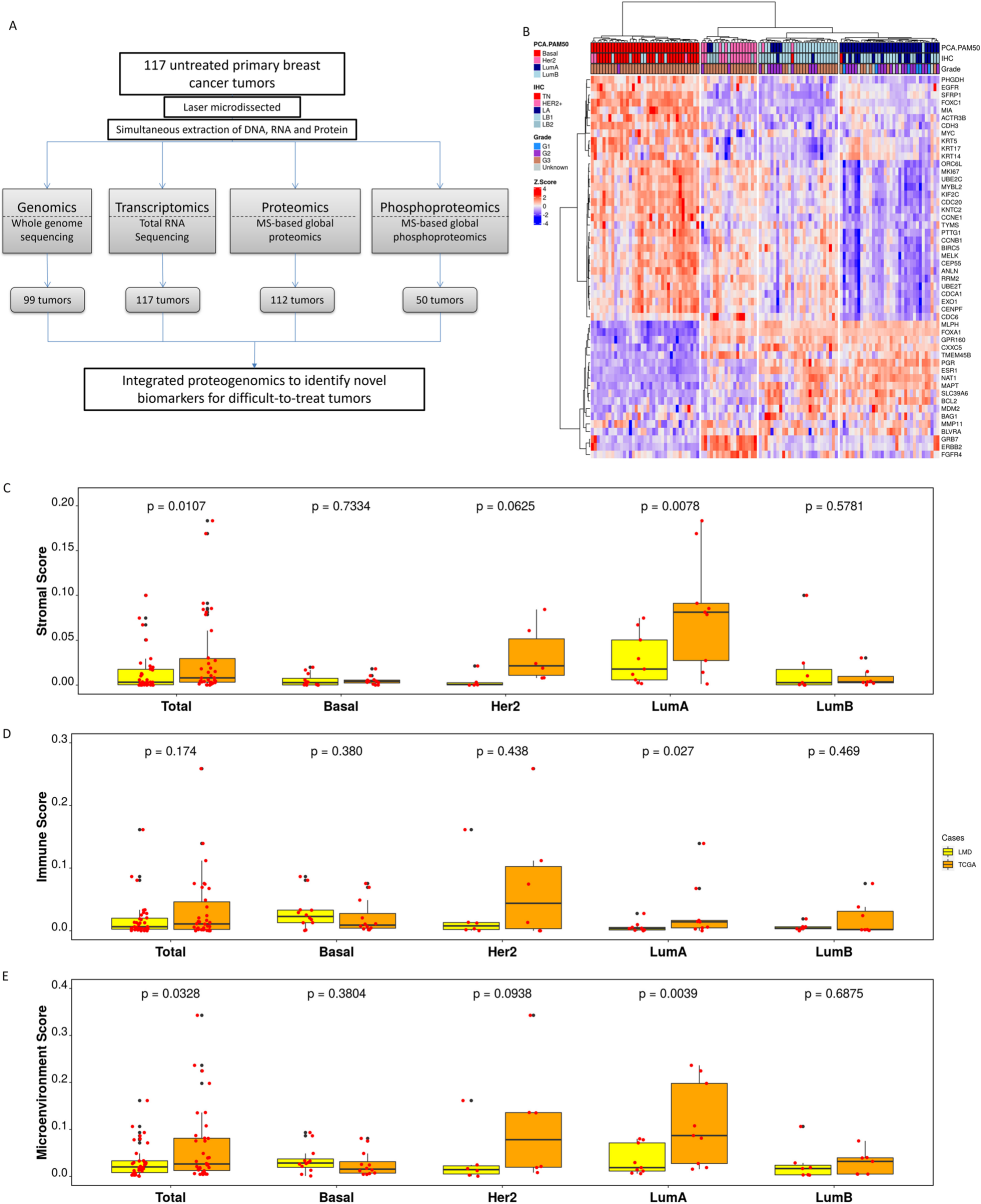
Overview of the study and the evaluation of LMD. (**A**) Overview of the number of cases used for each omics study. (**B**) Unsupervised clustering of the normalized PAM50 gene expression annotated with PCA-PAM50 and IHC subtypes. (**C-E**) Boxplot comparisons of stromal score (**C**), immune score (**D**) and microenvironment score (**E**) for all cases (Total) and stratified by subtype as inferred by xCell for the 34 cases that had RNA-seq data from both LMD (this study, yellow) and bulk processing (TCGA, orange). P-values were derived by pairwise Wilcoxon rank-sum tests

A total of 117 retrospectively collected, untreated primary breast tumor specimens were chosen for proteogenomic profiling with an emphasis on DTBC subtypes. We used IHC subtyping to enrich our cohort for DTBC tumors, resulting in over 87% of the tumors being non-LA: 30 TN, 16 HER2, 39 LB1, and 17 LB2. Additionally, we included 15 LA cases as a reference. We selected large (> = 1.5cm) tumors in our cohort to supply sufficient material for analysis of the same sample on multiple experimental platforms. An overview of the study design is presented in Fig. 1A, and the clinicopathologic characteristics of the cohort are presented in Table S1A.

Intrinsic subtypes of the tumors were derived using PCA-PAM50 [9] which enhanced the consistency with IHC subtyping (Table S1B and Table S1C). PCA-PAM50 classification is well reflected by the unsupervised clustering of the samples using the PAM50 genes (Fig. 1B). There were 78 (66.6%) DTBC tumors and 39 LumA tumors in our cohort, and the comparisons of their clinicopathologic characteristics are given in Table 1. This cohort, by design, had a much higher proportion of DTBC subtypes and differs from other major breast cancer studies like TCGA-BC study[29] and the Clinical Proteomic Tumor Analysis Consortium (CPTAC)-BC study[12, 13]. The two clinical variables significantly different between the two groups were patient age (*p*-value = 0.038) and grade (*p*-value < 0.001), which are well known prognostic factors for breast cancer. Tumors of DTBC subtypes had no significant differences in AJCC stage (*p*-value = 0.299) and tumor size (*p*-value = 0.4) to those of LumA subtypes, likely due to the selection of large tumors from all subtypes. This cohort had a long-term median follow-up of 9.2 years for DTBC and 9.3 years for LumA patients. The survival curves of the selected LumA cases were comparable to that of the DTBC cases for both overall survival (OS) and PFI (Fig. S1), in contrast to the general BC population where patients with DTBC tumors have worse clinical outcomes than those with LumA tumors [2, 68–70]. This discrepancy is likely due to the comparable stage and size between DTBC and LumA tumors in our cohort.

**Table 1:** Clinicopathologic characteristics for DTBC and LumA groups**.** Fisher's exact test was performed for the association of clinical features with the groups, and the p-value is included

Clinical features	DTBC (n = 78)	LumA (n = 39)	P-value
	N (%)	N (%)	
Patient age			
≤ 40	10 (12.8%)	2 (5.1%)	0.038
41–60	43 (55.1%)	15 (38.5%)	
> 60	25 (32.1%)	22 (56.4%)	
Race			
White	56 (71.8%)	35 (89.7%)	0.082
Black	15 (19.2%)	2 (5.1%)	
Other	7 (9%)	2 (5.1%)	
Menopausal status			
Pre-menopausal	29 (37.2%)	10 (25.6%)	0.278
Post-menopausal	39 (50%)	27 (69.2%)	
Surgically-menopausal	5 (6.4%)	2 (5.1%)	
Unknown	4 (5.1%)	0 (0%)	
Male	1 (1.3%)	0 (0%)	
Tumor grade			
G1	0 (0%)	6 (15.4%)	< 0.001
G2	10 (12.8%)	19 (48.7%)	
G3	66 (84.6%)	8 (20.5%)	
Unknown	2 (2.6%)	6 (15.4%)	
Tumor size			
T1	20 (25.6%)	13 (33.3%)	0.400
T2	51 (65.4%)	20 (51.3%)	
T3	5 (6.4%)	5 (12.8%)	
T4	1 (1.3%)	1 (2.6%)	
AJCC^a^ stage			
I	17 (21.8%)	9 (23.1%)	0.299
II	44 (56.4%)	21 (53.8%)	
III	17 (21.8%)	7 (17.9%)	
IV	0 (0%)	2 (5.1%)	
PFI^b^			
Event	15 (19.2%)	8 (20.5%)	1
Event-free	59 (75.6%)	29 (74.4%)	
Unknown	4 (5.1%)	2 (5.1%)	
Median follow-up in years	9.2	9.3	

DTBC, difficult-to-treat breast cancer; LumA, luminal A; N, number of cases; %, percentage out of total column; NA, not applicable

^a^AJCC, American Joint Committee on Cancer

^b^PFI, Progression free interval

In contrast to bulk processing, LMD processing of tumors enriches tumor cells and minimizes the contributions of stromal components which could vary from sample to sample. There were 34 cases in our cohort that were also part of the TCGA-BC study where bulk processing was used to prepare samples for DNA and RNA extraction. This enabled us to directly compare the effect of tissue processing methods on stromal contribution as measured by stromal score and immune score. When comparing all cases together regardless of subtype, significantly lower stromal and microenvironment (cumulation of stromal and immune) scores were observed in LMD-prepared samples compared to bulk-processed TCGA samples (Fig. 1C, E). When stratified by subtype, however, the stromal and microenvironment scores were only significantly lower in LMD samples of the LumA subtype. Interestingly, the immune score was also significantly lower in LumA LMD samples (Fig. 1D). The stromal and microenvironment scores differed to a lesser degree in the Her2 cases (Fig. 1C, E). It is important to note that the sample-to-sample variability is minimal in LMD. For example, the total stromal score Interquartile Range (IQR) for LMD is 0.017 and that of TCGA is 0.026. Consistent with the observation of score differences, there is reduced expression of stromal and immune-specific genes (*EDN3*, *GRIA4*, *WIF1* and *FDCSP*) in the LMD LumA cases compared to that of TCGA (Fig. S2 and Table S1D). Furthermore, immune-related pathways, such as allograft rejection, interferon gamma and alpha response, inflammation response, and complement cascade pathways were down-regulated in LMD LumA cases compared to that of TCGA (Table S1E).

### Mutational landscape assessment identifies enriched point mutations and structural variations as well as higher tumor mutational burden in DTBC tumors

Among all samples, WGS analysis identified 295,903 somatic single nucleotide variants (SNVs), 34,499 somatic insertions/deletions (INDELs), eight and ten significant chromosome arm-level amplification and deletion peaks, respectively, and 570 and 234 significant gene-level somatic copy number amplification and deletion peaks, respectively. There was a total of 10,393 somatic short variants (SNV and INDEL) impacting 5899 protein-coding genes.

**Fig. 2:**
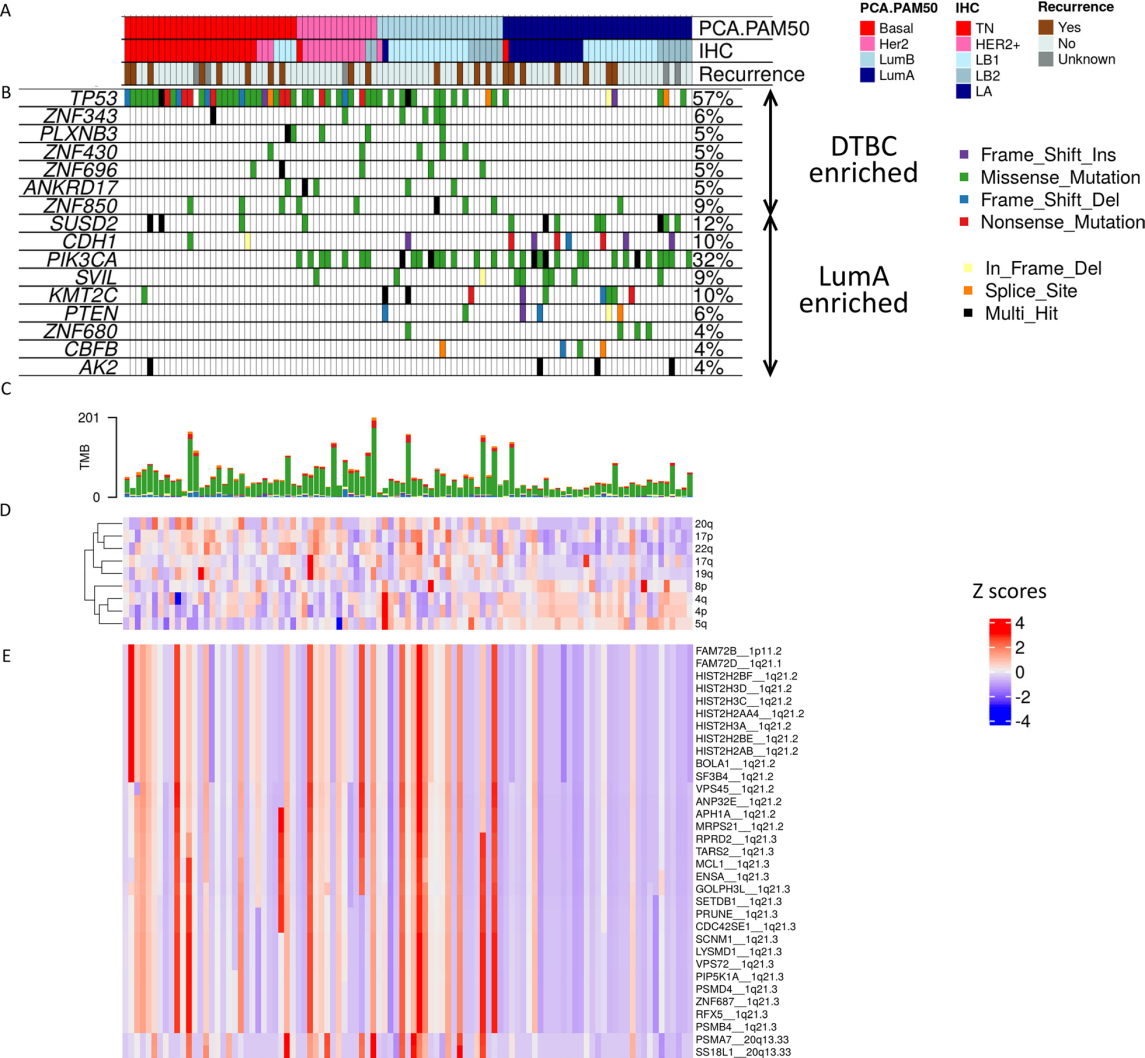
Mutational landscape of DTBC tumors in reference to LumA tumors. (**A**) Subtypes and recurrence status of tumors in top panel are sorted by DTBC and then LumA subtypes. (**B**) Oncoplot displaying the significantly mutated genes (SMG) enriched in DTBC tumors. Types of mutations in each gene are shown by a colored cell. Mutation frequency is shown on the right for each gene. Genes are ordered by their enrichment (DTBC versus LumA) and then sorted by p-value. (**C**) Tumor mutational burden (TMB) reported in terms of absolute non-synonymous mutations. (**D**) Significant chromosome arm level amplification and deletion peaks differentially enriched between DTBC and LumA tumors. (**E**) Somatic copy number alterations (SCNA)-based SMG differentially enriched between DTBC and LumA tumors

To identify somatic mutational events enriched in DTBC tumors, we compared the WGS-based somatic short variants (SNV/INDEL), large somatic copy number alterations (SCNA), chromosome arm level alterations, and tumor mutational burden (TMB) between DTBC and LumA tumors (Fig. 2). TMB was significantly (*p* < 0.001) higher in DTBC tumors compared to LumA tumors (Fig. 2C). There were 16 differentially mutated genes across the two groups, 7 and 9 of them were enriched in DTBC and LumA, respectively (Table S2A). Among the genes that showed significant enrichment of short variants in DTBC over LumA tumors was *TP53*, a well-documented tumor suppressor gene [71]; it was mutated in 76% of DTBC tumors compared to only 18% of LumA tumors (Fig. 2B and Table S2A). Interestingly, while recurrence rate for the two groups were the same (21%), 12 of the 50 DTBC tumors (24%) with *TP53* gene mutations had recurrences whereas 3 out of 6 LumA tumors (50%) with *TP53* gene mutations had recurrence (Fisher exact *p* = 0.33; Fig. 2A).

The other genes with enriched mutations in DTBC tumors included a recently reported cell surface protein, *PLXNB3*, that was described to be associated with poor survival in TNBC[72]; the *ANKRD17* gene that plays an important role in nuclear import and is also a substrate of the cell cycle transition-associated protein *CDK2*[73]; and the four zinc finger protein genes (*ZNF343*, *ZNF430*, *ZNF696* and *ZNF850*) associated with transcriptional regulation (Fig. 2B and Table S2A).

Several other previously reported BC-related gene mutations were found to be enriched in the LumA tumors of our cohort, including *CDH1*, *PIK3CA*, *SVIL*, *KMT2C*, *PTEN* and *CBFB* (Fig. 2B and Table S2A)[12, 29, 74]. Additional mutations were identified in the transmembrane protein gene, *SUSD2* (Sushi Domain Containing 2), the zinc finger protein gene, *ZNF680*, and a cell cycle control kinase gene, *AK2* (Fig. 2B and Table S2A).

Furthermore, nine chromosomal arm level amplification and deletion events were observed to be significantly different between DTBC and LumA tumors (Fig. 2D and Table S2B). All of these arm level lesions were previously identified in the TCGA [29] and CPTAC [12, 13] BC cohorts. Most notably, the 5q deletion characteristic of basal-like breast cancer was observed in 61% of DTBC tumors (Fig. 2D and Table S2B). The other chromosome arm level deletion events significantly enriched in DTBC tumors are 4p (73%), 4q (62%), and 8p (62%). The chromosome arm level amplification events occurring in at least 50% of DTBC tumors were 20q (73%), 22q (50%), 17q (56%), and 19q (53%). Remarkably, 22q and 17p were deleted in 79% and 67% of LumA tumors and amplified in 50% and 36% of DTBC tumors, respectively.

The analysis of cytoband level focal alterations revealed five cytobands significantly amplified in DTBC tumors (1p11.2, 1q21.1, 1q21.2, 1q21.3, and 20q13.33; FDR < 0.05); expression levels of 33 genes within these cytobands were positively correlated with their SCNA levels (r ≥ 0.3; Fig. 2E and Table S2C). 30 out of the 33 genes were part of cytoband 1q21, a region frequently amplified in tumors, including multiple types of breast cancer [75, 76]. These 33 genes included 24 proliferation-associated genes and several others that have been previously implicated in BC, including PR repressor *APH1A* [76, 77] and cell state regulator *MRPS21* [78]. The list also included two vesicle trafficking genes, *VPS45* [79] and *VPS72* [80], two RNA binding genes, *RPRD2* [81] and *LYSMD1* [82], sodium channel associated *SCNM1* [80], chromatin remodeling complex *SS18L1* [83], and growth factor *TARS2* [84]. The 1q21 amplification in Basal-like is largely aligned with a previously reported study that utilized the TCGA-BC dataset [76]. Additionally, our study demonstrated that cytoband 1q21 amplification is prevalent in all DTBC-subtypes (≥ 70%).

### Proteomic data clustering identifies two DTBC clusters and one LumA cluster

**Fig. 3:**
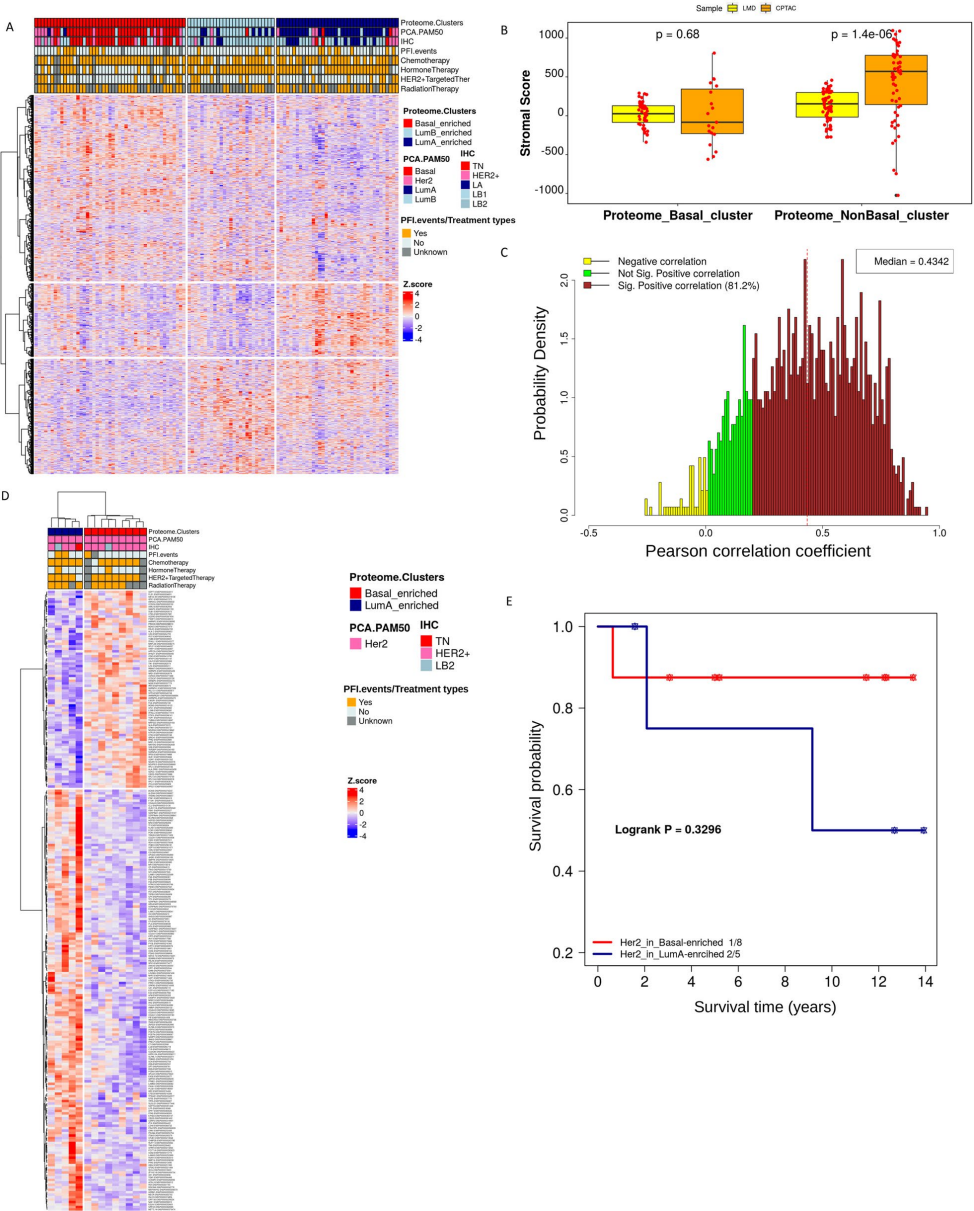
Proteomic clusters identify two DTBC clusters and one LumA cluster. **A** Hierarchical clustering of the consistently quantified 1,457 proteins where the column clustering is defined by K-means consensus clustering. **B** Comparison of ESTIMATE’s stromal score for the proteome clusters derived here and that of CPTAC-2016 using highly correlated (> 0.4) proteins. **C** mRNA:protein correlations using the co-quantified (no missing values) proteins and the common 1424 genes from RNA-Seq. **D** Hierarchical clustering of the significantly (FC > 1.2 and FDR ≤ 0.3) differentially expressed proteins between 9 Her2 of the Basal-enriched cluster and 5 Her2 of the LumA-enriched cluster. **E** The Kaplan–Meier curve of time to disease progression in years for Her2 cases in the Basal-enriched and LumA-enriched clusters for the endpoint, progression-free interval (PFI). P-value and the number of events/number of cases are given in the plot legends

MS-based global proteomics quantified (FDR < 0.01) a total of 5,898 distinct proteins (from 5634 genes) in at least one of the 112 cases. The 2735 proteins quantified in at least 70% of the cases were used for subsequent analyses. For clustering analysis, to minimize any potential bias from genes with multiple proteins, we selected the protein per gene with the highest variation, resulting in 1457 proteins. Unsupervised K-Means consensus clustering of these 1457 proteins identified 3 proteome clusters (Fig. 3 and Fig. S3). These clusters, according to the PCA-PAM50 subtypes, were classified as Basal-enriched (n = 47, 68% Basal), LumB-enriched (n = 27, 66.7% LumB), and LumA-enriched (n = 38, 71% LumA) (Fig. 3A and Table S3).

Unlike a recent report [13] on proteomics clustering of bulk-processed tumors, we did not observe a stromal-enriched cluster, probably because the use of LMD minimized stromal components. To examine this more comprehensively, we compared the stromal scores of our clusters to those of Mertins et al. [13] and found that stromal scores for LMD tumors in non-Basal clusters were significantly lower than that of Mertins, et al. 2016 (Fig. 3B). mRNA-protein correlation was used to assess the heterogeneity of the tumors[85]. The mRNA--protein correlation using the 1424 mRNA-protein pairs demonstrated a median Pearson correlation coefficient (r) of 0.43 (Fig. 3C), which is 11% higher than the median r observed in Mertins et al. 2016. To perform a more direct comparison, we conducted another correlation analysis on our data and the data from Mertins et al. 2016, using the 310 mRNA-protein pairs common between both datasets. We observed a median r of 0.63 with our data, which is 10% higher than the median r of 0.57 using the Mertins, et al. data (Fig. S3C & D). The higher correlation in our data could be due to the enrichment of tumor cells by LMD and the simultaneous extraction of RNA and protein from the same tissue preparation in our study.

We also observed that Her2 cases were mostly distributed across Basal-enriched and LumA-enriched protein clusters (Table S3 and Fig. 3A), as previously observed by Krug et al. [12] and Mertins, et al. [13]. To investigate if there is any clinical implication for the nine and five Her2 cases in the Basal-enriched and LumA-enriched clusters, respectively, we performed a survival and molecular analysis. There was no significant survival difference with PFI (*p* = 0.330; Fig. 3E). However, we observed 80 up-regulated and 171 down-regulated differentially expressed proteins (DEP; FC > 1.2 and FDR ≤ 0.3) in the Basal-enriched vs. LumA-enriched Her2 tumors. The unsupervised hierarchical clustering of those 251 DEPs captured the bifurcation of Her2 tumors in LumA-enriched and Basal-enriched clusters (Fig. 3D). Among these DEPs, five upregulated and two downregulated proteins in the basal-enriched cluster displayed changes in the same direction as the somatic copy number alteration (SCNA) and RNA expression levels (Fig. S4). Notably, the previously reported marker PGK1, predictive of poor survival in BC [86], exhibited upregulation in Her2 tumors associated with the LumA-enriched proteome cluster (Fig. S4B).

To explore whether any outcome differences may be coincident with different treatment regimens applied to different patients, the cases shown in Fig. 3 were annotated with types of treatments (Chemotherapy, Hormone therapy, HER2 + targeted therapy, and Radiation therapy). As shown in Fig. 3D, there is no observed difference in treatment among the Her2 cases from the two different clusters.

### Phosphoproteomic clustering analysis reveals Basal clusters with trended outcome differences

**Fig. 4:**
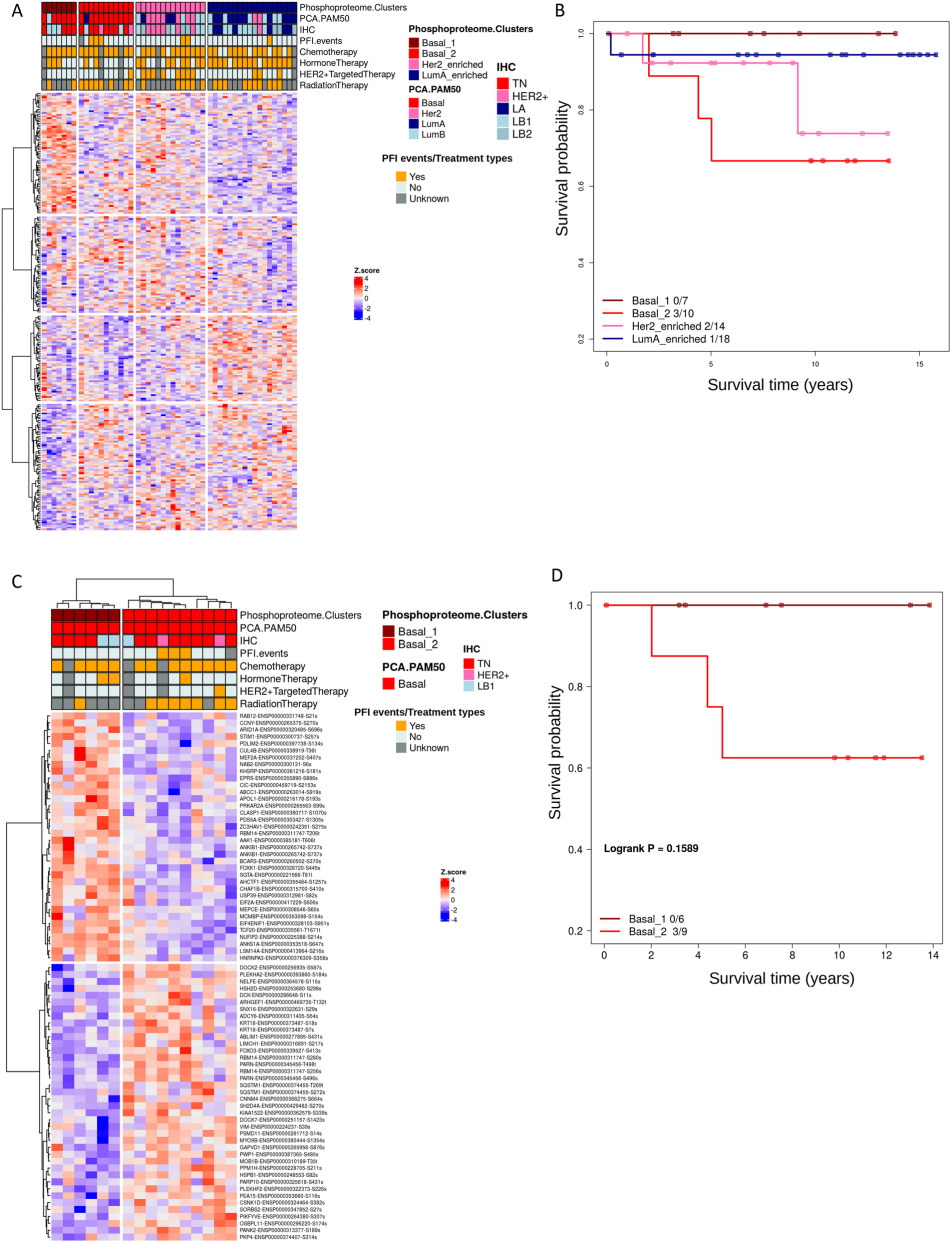
Phosphoproteomic clusters identify Basal clusters with trended outcome differences. **A** Hierarchical clustering of the consistently quantified 245 phosphosites where the column clustering is defined by K-means consensus clustering. Fig. S7 presents this figure with the names of phosphopeptides. **B** The Kaplan–Meier curves of time to disease progression in years for all four phosphoproteome clusters for the endpoint of progression-free interval (PFI). **C** Hierarchical clustering of the 76 significantly (FC > 1.2 and FDR ≤ 0.2) differentially expressed phosphopeptides between 6 Basal cases in the Basal_1 cluster and 10 Basal cases in the Basal_2 cluster. Phosphopeptides are included in the heatmap in the “GeneSymbol-ProteinEnsemblID-phosphosite” format. **D** The Kaplan–Meier curves of time to disease progression in years for Basal cases in the two Basal clusters for PFI. P-values and the number of events/number of cases are given in the plot legends

MS-based global phosphoproteomics quantified (FDR < 0.01) a total of 5049 phosphopeptides (from 2,093 proteins and 2,065 genes) in at least one of the 50 cases. The 331 phosphopeptides (from 245 genes) quantified in all of the cases for phosphoproteomics were used for differential expression analyses. For clustering analysis, to minimize any potential bias from genes with multiple peptides, we selected the phosphopeptide per gene with the highest variation, resulting in 245 phosphopeptides. Unsupervised K-Means consensus clustering using these 245 unique phosphopeptides resulted in 4 optimal clusters (Fig. 4 and Fig. S5), including two Basal-enriched clusters designated as Basal 1 (n = 7, 85.7% Basal) and Basal 2 (n = 11, 90.9% Basal), a Her2-enriched cluster (n = 14, 50% Her2), and a LumA-enriched cluster (n = 18, 55% LumA) (Fig. 4A, Fig. S7 and Table S4). We performed survival analyses of the 4 clusters using the endpoint of PFI and observed that although not statistically significant, the Basal 2 cluster had the worst survival, and surprisingly, the Basal 1 cluster had no PFI events (Fig. 4B).

We examined the differences among the Basal cases in more detail. We termed the 10 Basal cases in the Basal-2 cluster as the high relapse-risk group and the 6 cases in the Basal-1 cluster as the low relapse-risk group. The differential expression analysis between the two groups, with all of the 331 quantified phosphopeptides, identified 40 and 36 significantly (FC > 1.2 and FDR ≤ 0.2) up-regulated and down-regulated phosphopeptides, respectively, in the Basal-2 versus Basal-1 clusters. The unsupervised hierarchical clustering of these 76 phosphopeptides captured the distinct profiles of the two Basal groups (Fig. 4C). There was also a trending PFI difference between the two Basal clusters (*p* = 0.16; Fig. 4D).

**Fig. 5:**
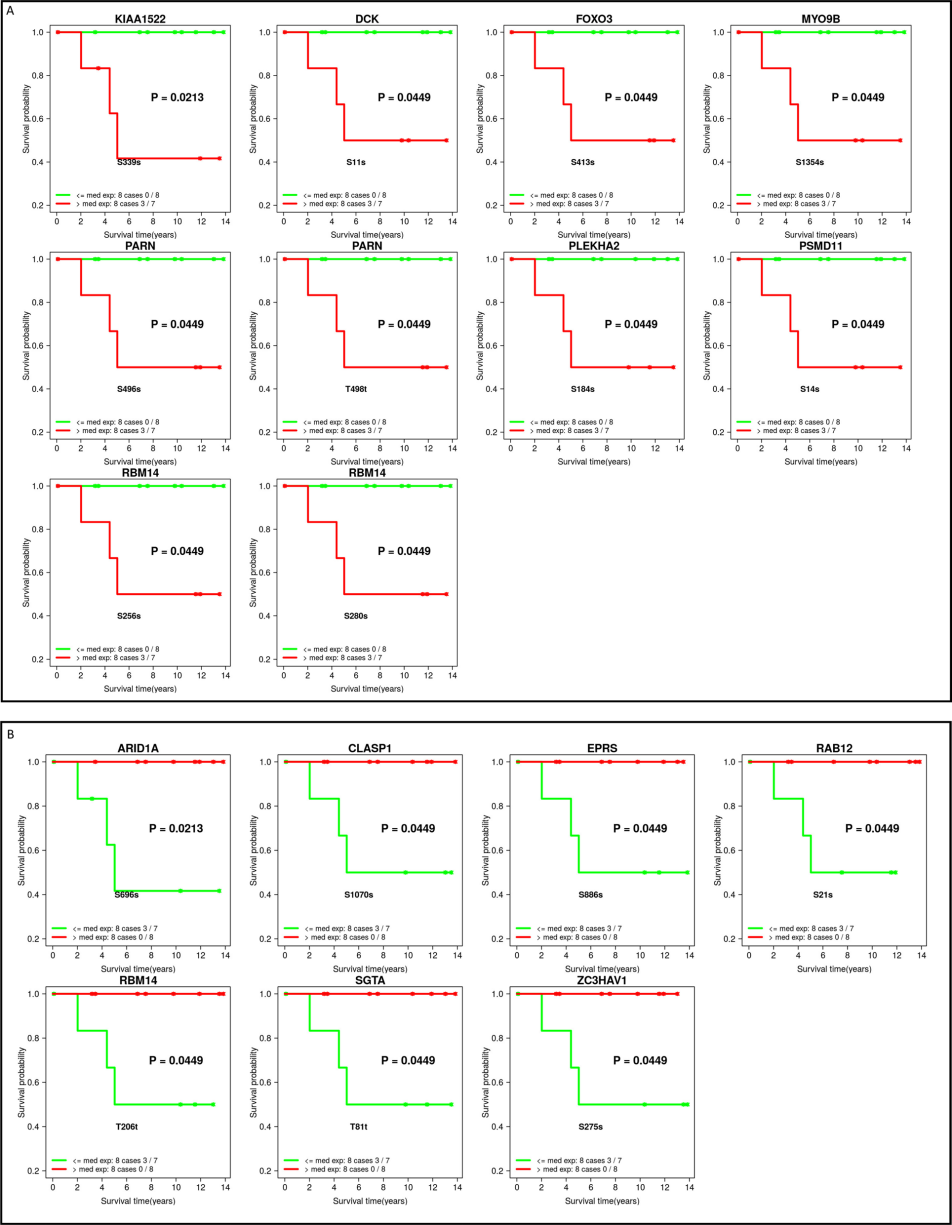
Phosphopeptide expression significantly associated with Basal disease progression. Kaplan–Meier curves of time to disease progression (PFI) in years for the significantly (FC > 1.2 and FDR ≤ 0.2) differentially expressed phosphopeptides between 10 Basal cases of the Basal_2 cluster (high relapse-risk) and 6 Basal cases of the Basal_1 cluster (low relapse-risk). High (> median) expression of 10 up-regulated (**A**) and low (< median) expression of 7 down-regulated (**B**) phosphopeptides, respectively, in the Basal_2 cluster that were significantly (*p* < 0.05) associated with a worse progression free interval (PFI). Gene name, *P*-value, phosphosite and the number of events/number of cases are given in each plot

To explore potential markers of survival outcome differences, we tested each of the 76 phosphopeptides, using median separation of expression, for its ability to separate Basal cases into high relapse-risk and low relapse-risk groups. Most of the 76 differentially expressed phosphopeptides, by their high (> median) and low expression (≤ median), provided at least a trending separation of high relapse-risk and low relapse-risk Basal cases (Fig. S6). Importantly, 17 phosphopeptides (10 up-regulated and 7 down-regulated; representing 14 genes) were able to significantly distinguish high relapse-risk cases from the low relapse-risk cases (log rank *p* < 0.05; Fig. 5 and Table 2). Notably, among the 17 phosphopeptides, three were from the gene *RBM14*, two of which were up-regulated (sites S280s and S256s) and one that was down-regulated (site T206t) in the high relapse-risk group (Fig. 5 and Table 2). Many of the 14 genes represented by the 17 phosphopeptides identified here have been previously reported to play significant roles in breast cancer, including *KIAA1522*, *DCK*, *FOXO3*, and *MYO9B* among the 8 up-regulated genes [87–90] and *ARID1A*, *EPRS*, and *ZC3HAV1* among the 7 down-regulated genes [91–93] in the high relapse-risk cases.

**Table 2:** Differentially expressed phosphopeptides in high relapse-risk and low relapse-risk Basal cases

Gene Symbol	Ensembl Protein ID	Phosphorylation site	Description	Log-rank p-value
Up-regulated in Basal_2 cluster (high relapse-risk BC)				
KIAA1522	ENSP00000362579	S339s	KIAA1522	0.021
DCK	ENSP00000286648	S11s	Deoxycytidine kinase	0.045
FOXO3	ENSP00000339527	S413s	Forkhead box O3	0.045
MYO9B	ENSP00000380444	S1354s	Myosin IXB	0.045
PARN	ENSP00000345456	S496s	Poly-A specific ribonuclease	0.045
PARN	ENSP00000345456	T498t	Poly-A specific ribonuclease	0.045
PLEKHA2	ENSP00000393860	S184s	Pleckstrin homology domain containing A2	0.045
PSMD11	ENSP00000261712	S14s	Proteosome 26S Subunit, non-ATPase 11	0.045
RBM14	ENSP00000311747	S256s	RNA-binding motif protein 14	0.045
RBM14	ENSP00000311747	S280s	RNA-binding motif protein 14	0.045
Down-regulated in Basal_2 cluster (high relapse-risk BC)				
ARID1A	ENSP00000320485	S696s	AT-rich interaction domain 1A	0.021
CLASP1	ENSP00000380717	S1070s	Cytoplasmic linker associated protein 1	0.045
EPRS	ENSP00000355890	S886s	Glutamyl-prolyl-tRNA synthetase 1	0.045
RAB12	ENSP00000331748	S21s	RAB12, member RAS oncogene family	0.045
RBM14	ENSP00000311747	T206t	RNA-binding motif protein 14	0.045
SGTA	ENSP00000221566	T81t	Small glutamine rich tetratricopeptide repeat co-chaperone alpha	0.045
ZC3HAV1	ENSP00000242351	S275s	Zinc finger CCCH-type containing, antiviral 1	0.045

The 17 phosphopeptides that demonstrated significant survival differences (p < 0.05) using the progression-free interval (PFI) between Basal_2 cluster (n = 10) and Basal_1 cluster (n = 6) in Fig. 4C

To further investigate whether any outcome differences may be influenced by the different treatments the patients received, the cases shown in Fig. 4 were annotated with types of treatments. As shown in Fig. 4C, there were no observed treatment differences among the Basal cases in the two different groups (Fisher exact test *p* = 1.0).

### Pathway analysis reveals strong enrichment of proliferation-associated pathways in DTBC tumors

Differential expression analysis identified 2,077 differentially expressed genes (402 upregulated and 1675 downregulated with |FC|> 2 and an adjusted *p*-value < 0.01) and 189 differentially expressed proteins (62 upregulated and 127 downregulated with |FC|> 1.2 and an adjusted p-value < 0.05) between DTBC and LumA tumors. However, only 55 genes/proteins (11 upregulated and 44 downregulated in DTBC) were common between the significantly differentially expressed genes and proteins (Table S5A).

**Fig. 6:**
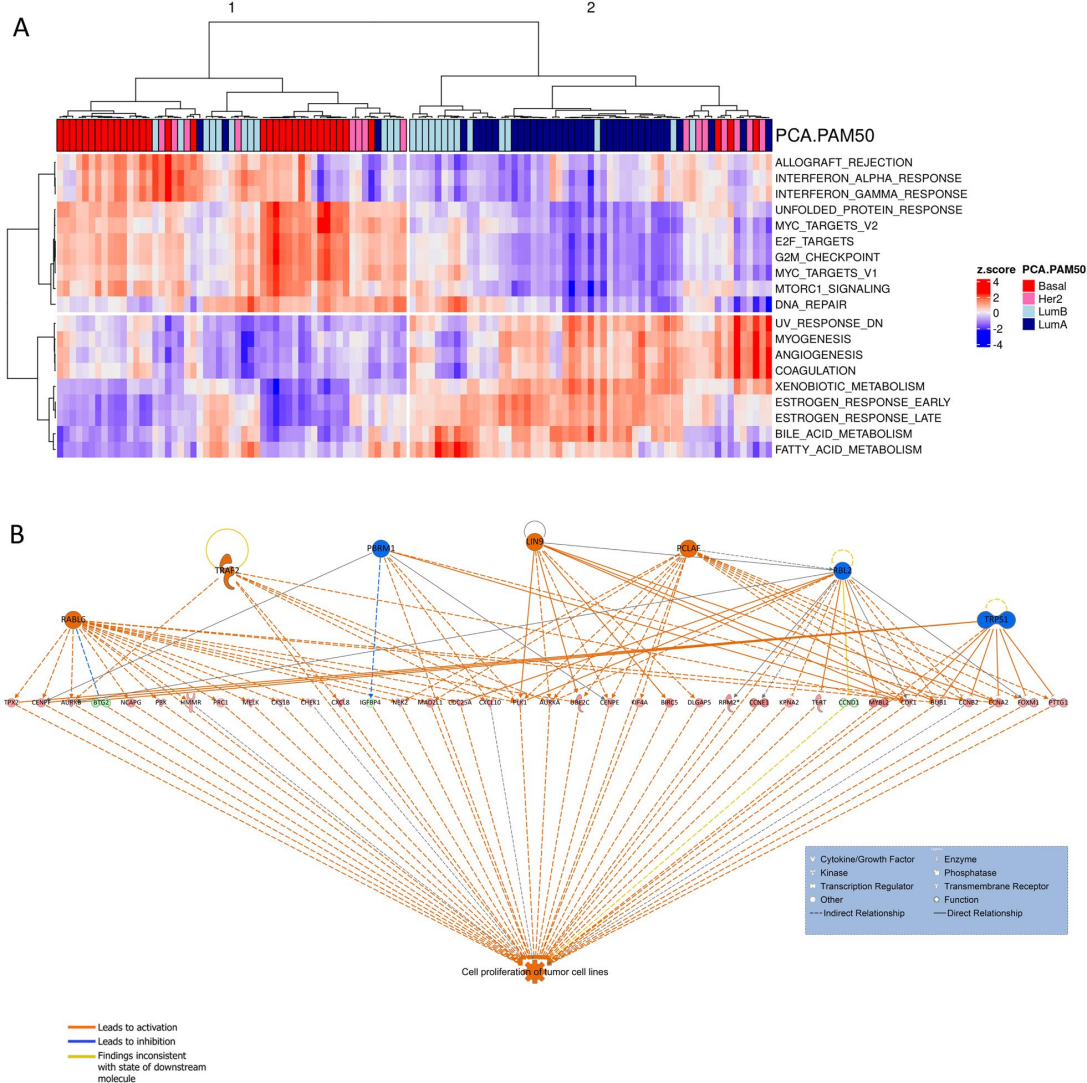
Pathways and activated functional regulatory networks. **A** The unsupervised hierarchical clustering of the 19 significant pathways based on their gene set enrichment scores identified from multi-omics gene-set analysis (MOGSA). **B** The top activated network identified using RNA-Seq data from Ingenuity pathway analysis (IPA)

To investigate the molecular and pathway differences between DTBC and LumA tumors, we employed Multi-Omics Gene-Set Analysis (MOGSA) [66], Gene Set Enrichment Analysis (GSEA), and Ingenuity Pathway Analysis (IPA) [67]. MOGSA, a method for single-sample gene set enrichment analysis integrating all transcriptomic and proteomic features, identified 19 significant pathways (10 upregulated and 9 downregulated in DTBC, Table 3 and Fig. 6A). Among the 19 pathways, 16 were confirmed by GSEA using either transcriptomic or proteomic data independently (Table 3).

**Table 3:** The 19 hallmark pathways identified by multi-omics gene-set analysis (MOGSA) using both RNA-Seq and proteomics data that are significantly (GLM p < 0.01) differentially regulated between DTBC and LumA

Gene.Set	MOGSA GLM *p*.value	GSEA Transcript FDR	GSEA Protein FDR	Direction MOGSA	Direction GSEA transcripts	Direction GSEA proteins	Process category
MTORC1_SIGNALING	< 0.001	< 0.001	< 0.001	Up	Up	Up	Signaling/Proliferation
E2F_TARGETS	< 0.001	< 0.001	< 0.001	Up	Up	Up	Proliferation
G2M_CHECKPOINT	< 0.001	< 0.001		Up	Up	Up	Proliferation
UNFOLDED_PROTEIN_RESPONSE	< 0.001	0.001	< 0.001	Up	Up	Up	Pathway
MYC_TARGETS_V1	< 0.001	< 0.001	< 0.001	Up	Up	Up	Proliferation
MYC_TARGETS_V2	< 0.001	< 0.001	0.013	Up	Up	Up	Proliferation
DNA_REPAIR	< 0.001	0.004		Up	Up		DNA damage
INTERFERON_ALPHA_RESPONSE	< 0.001	< 0.001	0.003	Up	Up	Up	Immune
INTERFERON_GAMMA_RESPONSE	< 0.001	< 0.001	0.001	Up	Up	Up	Immune
ALLOGRAFT_REJECTION	< 0.001	< 0.001	< 0.001	Up	Up	Up	Immune
XENOBIOTIC_METABOLISM	< 0.001		0.014	Down		Down	Metabolic
ESTROGEN_RESPONSE_EARLY	< 0.001	< 0.001	0.005	Down	Down	Down	Signaling
ESTROGEN_RESPONSE_LATE	< 0.001	0.001	< 0.001	Down	Down	Down	Signaling
BILE_ACID_METABOLISM	< 0.001			Down			Metabolic
UV_RESPONSE_DN	< 0.001	0.004	< 0.001	Down	Down	Down	DNA damage
MYOGENESIS	< 0.001	0.002	0.014	Down	Down	Down	Development
ANGIOGENESIS	0.001			Down			Development
COAGULATION	0.002		0.002	Down		Down	Immune
FATTY_ACID_METABOLISM	0.003			Down			Metabolic

GSEA using RNA-Seq and proteomics data that agree in direction of up and down-regulation in DTBC versus LumA

These 19 pathways were further annotated by their biological process category [65, 94] (Table 3). Among the 10 upregulated pathways, 5 belonged to the proliferation category (MTORC1 signaling, E2F targets, MYC targets V1 and V2, and the G2M checkpoint). The other 5 upregulated pathways belonged to immune response (Interferon Alpha and Gamma response, Allograft rejection), DNA damage response (DNA repair), and an unclassified category (Unfolded protein response). In contrast, LumA tumors showed enrichment in metabolism pathways (Xenobiotic metabolism, Bile acid metabolism, Fatty acid metabolism), Signaling (Estrogen response early and late), Development (Myogenesis, Angiogenesis), DNA damage response (UV response downregulated), and Immune response (Coagulation) (Table 3).

The IPA core pathway analysis, using significantly differentially expressed features, reports the top activated functional network(s). From differentially expressed genes, Cell proliferation of the tumor was identified as the top activated functional network, with 36 differentially expressed genes connected to 7 known regulators (Table S5B; Fig. 6B). From the differentially expressed proteins, the FOXC1 regulatory network was identified as the top activated functional network, with 10 differentially expressed proteins involved in this network (Table S5C). *FOXC1*, an emerging oncogene, is associated with cell progression, proliferation, differentiation, and metastasis [95]. From differentially expressed phosphopeptides, Cellular assembly and organization was revealed as the top function (Table S5D). A common theme of the networks identified by IPA from the three different datasets is that they are all involved with cell proliferation. These findings are also corroborated by the enrichment of proliferation-associated pathways and the amplification of proliferation-associated genes identified by the MOGSA and SCNA analyses, respectively (Figs. 2E and 6B).

## Discussion

We performed proteogenomic profiling of a cohort enriched for DTBC subtypes after laser microdissection with simultaneous extraction of DNA, RNA and protein. The DTBC group, in reference to the LumA group, includes patients of younger age, black race and with higher-grade tumors. However, there is no significant difference in tumor stage and size between the two groups, probably due to the selection of larger-sized tumors for the study. Our study revealed additional complexities in the heterogeneity of breast cancer, reiterating the value of proteogenomic integration in uncovering novel targets for cancer and potential therapeutic interventions.

Laser microdissected tumor samples showed significantly lower stromal, immune and microenvironment scores in non-Basal tumors, especially LumA tumors, compared to that of bulk-processed breast tumors from TCGA (Figs. 1C–E and 3). There was also significantly lower stromal gene expression in LMD LumA tumors compared to TCGA LumA tumors (Fig. S2). Furthermore, in contrast to the 2016 CPTAC study [13], no stromal-enriched cluster was observed, and the correlation between mRNA-protein was higher in our cohort. These results indicate that bulk-processed samples probably contain a varied mixture of stromal cells and malignant epithelial cells, complicating the interpretation of proteogenomic profiles. A pertinent reported instance involves TCGA-Glioblastoma data derived from reverse-phase protein arrays, where the use of whole sections led to misinterpretation by certain researchers, erroneously indicating elevated PTEN expression within the tumor, whereas subsequent research employing LMD demonstrated that the dominant signal was from non-tumor cells [96]. Similarly, our study also emphasizes the importance of using laser microdissection to investigate the distinct biology of enriched cancer cells separately from surrounding stromal cells.

Our mutation analysis revealed many features differentially enriched between the DTBC vs LumA tumors, with many implicated in BC previously, such as TP53 mutations, 5q deletion, 1q21 amplification, etc. Our study demonstrated that many of these features are shared among DTBC tumors. This could suggest a potentially common cell-of-origin for DTBC tumors, such as an ER-negative cancer stem cell or a progenitor cell for these tumor subtypes. The proliferation-associated genes within 1q21 (Table S2C) should be investigated further to determine if they could be potential therapeutic targets.

Strong enrichment of cell proliferation-associated pathways was observed in DTBC tumors from multiple analyses (Fig. 6, Table 3, and Table S5). For example, the E2F transcription factor targets pathway, a well-known key regulator of cell proliferation [97, 98], was highly significant. This pathway involves key molecules like *CDKN3* which was also overexpressed in DTBC tumors (Fold change > 2; adj. *p*-value < 0.001). Overexpression of *CDKN3* is reported as a predictor of poor survival and promotor of proliferation and migration in many cancers including BC [73, 99–102]. In addition, pathway analyses using significantly differentially expressed proteins predicted activation of the FOXC1 network. *FOXC1* is an emerging oncogene and known to be involved in cell progression, proliferation, differentiation, and metastasis [95]. This phenomenon could potentially explain why DTBC tumors are highly proliferative and aggressive tumors in general [68, 70, 103]. Given that our study used tumors of comparable stages and sizes, we can ascertain that enrichment of proliferation pathways in DTBC tumors is more related to the molecular differences among subtypes than due to differences in tumor stage or size.

Proteomics-based clustering identified two DTBC-specific clusters (Basal-enriched and LumB-enriched) and a LumA-enriched cluster. In accordance with previous proteogenomic studies [12, 13], Her2 cases were split between the Basal-enriched and LumA-enriched clusters and exhibited differences in protein expression (Fig. 3D and Table S3). Most notably, high expression of phosphoglycerate kinase 1 (PGK1) was seen among Her2 cases in the LumA-enriched cluster. Previous studies have reported that high expression of PGK1 is associated with worse survival especially in Her2 cases [86, 104]. The role of high PGK1 expression in this subset of Her2 cases could be further explored as a potential therapeutic marker.

The integration of long follow-up outcome data with the phosphoproteomic clusters enabled the identification of phosphoproteomic profile differences between high relapse-risk and low relapse-risk Basal BC. The successes of using phosphoproteomic profiles to separate Basal cases was also reported previously by Zagorac, et al. [105]. Two of the 14 genes represented by the 17 phosphopeptides, *RBM14* and *MYO9B*, were also reported in the Zagorac, et al. study, although they observed phosphorylation at a different phosphosite.

Many of the 14 genes we identified have been previously reported to play significant roles in breast cancer and other cancers, reinforcing the importance of our phosphopeptide discovery. Among the 10 phosphopeptides upregulated in the high relapse-risk group, *KIAA1522*, *DCK*, *FOXO3* and *MYO9B* are notable for their association with aggressive cancer phenotypes. *KIAA1522*'s elevation in triple-negative breast cancer tissues has been reported for its oncogenic potential and role in promoting visceral metastasis [87]. The *DCK* gene, known for its increased expression in breast cancers with poor prognosis [88], is associated with the action of Decitabine, an FDA-approved drug for certain blood cancers [106], which has also been shown to significantly inhibit the growth of triple-negative breast cancer [107]. *FOXO3* has been implicated in the coordinated increases in glycolysis and apoptosis resistance in TNBC and proposed as an attractive therapeutic target for TNBC [90]. High levels of *MYO9B* have been shown to promote actin reorganization by reducing filaments and to stimulate metastasis by breaking down stress fibers and reducing cell adhesion, thereby enhancing the cancer phenotype in both prostate [89] and lung cancer [108].

The downregulation of phosphopeptides in genes like *ARID1A*, *EPRS*, and *ZC3HAV1* in the high relapse-risk breast cancer group offers critical insights into their roles as tumor suppressors and regulatory molecules. The downregulation of *ARID1A*, known for its potential in DNA repair and immune response modulation, in triple-negative breast cancer, marks it as a target for immune checkpoint inhibitors [91]. *EPRS* was reported as a critical regulator of cell proliferation and estrogen signaling in ER + breast cancer [109] and has also been implicated as a potential treatment target for basal-like breast cancer [92]. ZC3HAV1, a PARP family enzyme, promotes proliferation and metastasis by regulating KRAS in pancreatic cancer [110] and is involved in facilitating DNA repair and promoting tumorigenesis in breast cancer [93].

It is noteworthy that we observed three phosphopeptides from RBM14 with different directions of differential expression. *RBM14* is known to function in transcription and RNA splicing; different isoforms are encoded by alternatively spliced transcript variants and have been reported to have opposing effects on transcription [111]. The different directions of enrichment of the three RBM14 phosphopeptides in our study indicate that there may be coordinated or opposing regulation among the different phosphorylation sites to carry out the different functions of this important protein. RBM14 is known to physically interact with PARP1, which is a key player in the DNA damage response (DDR) network and a target of cancer therapy [112]. RBM14 has been implicated in the migration of breast cancer [113], heightened radio-resistance in glioblastoma [114], and more recently, promoting cell growth in lung cancer [115].

Protein phosphorylation plays a crucial role in activating and deactivating complex regulatory networks. Therefore, the directionality of the differential enrichment of phosphopeptides observed in our study should not be straightforwardly interpreted as being associated with either the activation or suppression of tumorigenesis or progression. Further investigation is needed for all 17 differentially expressed phosphopeptides (Fig. 5) to determine their potential as biomarkers. This could help distinguish between more and less aggressive forms of Basal breast cancer and potentially guide treatment decisions.

Treatment selection is critical for cancer therapy and long-term outcome. Although in our analysis, the Basal subgroups of patients in our cohort didn’t receive different treatments, they were separated into subgroups with different relapse risks by the phosphopeptide features. This finding suggests the potential use of these phosphopeptides as biomarkers for improved personalized therapy, such as less aggressive treatment for patients in the low relapse-risk group.

This study has two limitations. First, the use of large tumors may not fully represent the broader tumor population of different sizes, thus, caution needs to be exercised when extrapolating the findings made in our study to tumors of smaller size. Second, our study focused on tumor-enriched cells; however, to comprehend how a tumor acts in vivo, it is imperative to study tumor cells as well as stromal cells. While it would be ideal to study purified tumor and stromal cells simultaneously or purified tumor cells paired with the whole section (including stroma and other cell types), the demand for tissue either way is much higher. Limited by the available resources, our study was designed to focus on the tumor only. As technologies improve to require significantly less DNA/RNA/Protein, the challenges we are facing for the simultaneous analysis of tumor and stromal components will be eased.

Taken together, we have shown that LMD provides advantages for BC research and that DTBC tumors possess similar aggressive, molecular properties. In addition, we have identified potential molecular markers for predicting outcomes for patients with less responsive basal-like tumors. In conclusion, integrating molecular data from different platforms and conducting orthogonal computational methods has provided new insights into breast cancer subtypes and has also contributed to identifying potential drug targets for the difficult-to-treat basal-like subtype of breast cancer.

### Supplementary Information

Additional file 1. Figure S1. Kaplan–Meier Curves for the Cohort Based on PCA-PAM50 Subtypes and DTBC and LumA Subtype groups. Panels (A) and (B) display Kaplan–Meier curves for cumulative survival in years across PCA-PAM50 subtypes. Panels (C) and (D) present Kaplan–Meier curves for cumulative survival for DTBC and LumA subtypes. The endpoint of overall survival is used for panels (A) and (C) while the endpoint of progression-free interval is used for panels (B) and (D). The legends of the plots include the p-value and the count of events/total cases.

Additional file 2. Figure S2. Unsupervised clustering of 9 LumA samples, a subset of the 34, using differentially expressed genes between LMD (yellow) and bulk processing (TCGA, orange). Histology, Stromal score, Immune score and Microenvironment score are provided as annotation. The corresponding pairs of LMD and TCGA samples were suffixed as P1, P2, etc

Additional file 3. Figure S3. Quality metrics of K-means clustering and correlation analysis of the proteomics data. (A) Visualization of consensus matrices from K-means consensus clustering for K = 2, 3 and 4. (B) Silhouette plots are shown for K = 2, 3 and 4 clusters to evaluate the coherence of the clustering. K = 3 was selected as the optimal cluster because of its better separation and silhouette width. (C) The mRNA:protein correlations for 310 proteins overlapping between the LMD and CPTAC-2016 (D) datasets.

Additional file 4. Figure S4. Multi-omics differences between Her2 cases of the Basal-enriched versus Luminal A-enriched protein clusters. Significantly up-regulated (A) and down-regulated proteins (B) in Her2 cases of the Basal-enriched versus Luminal A-enriched protein clusters, which also shows significant up and down-regulation, respectively, in the other two omics (transcriptomics (RNA) and genomics (SCNA)). Wilcoxon rank sum test p-value is given in the plot where p < 0.05 is considered significant. In the case of non-significant SCNA difference, the trended difference (p < 0.2) is included.

Additional file 5. Figure S5. Quality metrics of K-means clustering of phosphoproteomics. (A) Visualization of consensus matrices from K-means consensus clustering for K = 2, 3, 4 and 5. (B) Silhouette plots are shown for K = 2, 3, 4 and 5 clusters to evaluate the coherence of the clustering. K = 4 was selected as the optimal cluster for its better separation and non-negative silhouette width.

Additional file 6. Figure S6. Kaplan–Meier curves of time to disease progression in years for the significantly (FC > 1.2 and FDR ≤ 0.2) differentially expressed phosphopeptides between 10 basal cases of the Basal_2 cluster (high relapse-risk) and 6 basal cases of the Basal_1 cluster(low relapse-risk). The high (> median) and low (< median) expression of all 40 up-regulated (A) and 36 down-regulated (B) phosphopeptides in the Basal_2 versus Basal_1 clusters with the end point of PFI. Gene name, P-value, phosphosite and the number of events/number of cases are given in each plot.

Additional file 7. Figure S7. Phosphoproteomic clusters with the names of Phosphopetides. This hierarchical clustering is identical to Fig. 4A but includes the names of all 245 phosphopeptides in the “GeneSymbol-ProteinEnsemblID-phosphosite” format.

Additional file 8. Table S1. Subtypes and Gene set enrichment analysis. (A) Contingency table comparing PCA-PAM50 and IHC subtypes. (B) Annotation of the differentially expressed genes between LMD and TCGA LumA samples. (C) Gene Set Enrichment Analyses highlight sets of immune-related pathways significantly down-regulated in LMD LumA tumors.

Additional file 9. Table S2. Differential mutations and SCNA between DTBC and LumA tumors. (A) Significantly (*p*-value < 0.1) differentially mutated genes with non-synonymous somatic short variants (SNV and INDEL) in DTBC versus LumA tumors. Odds ratio and p-value of the Firth logistic regression are reported. (B) Somatic copy number alterations (SCNAs) at the chromosome arm level that show significant differences (*p* < 0.05) between DTBC and LumA tumors. Samples with a value of >  = 0.1 were classified as amplified, and those with <  = − 0.1 were categorized as deleted. The table’s order corresponds to the clustering arrangement of Fig. 2D. (C) Genes linked with focal SCNA peaks displaying significant differences (FDR < 0.05) between DTBC and LumA tumors. Cases with a relative SCNA of >  = 0.1 are categorized as amplified, while those with <  = − 0.1 are classified as deleted. The table provides Wilcoxon test p-values, adjusted p-values, and Pearson’s correlation coefficient (r) for gene’s SCNA and RNA expression. The table is arranged by cytoband, start coordinate, and FDR-adjusted *p*-values. Genes associated with cell proliferation are indicated in the table.

Additional file 10. Table S3. Contingency table comparing mRNA-derived PCA-PAM50 subtypes to proteome clusters. The majority of the PCA-PAM50 subtype in each proteome cluster is highlighted in green. The Her2 subtype separated in the Basal-enriched and LumA-enriched clusters is highlighted in red.

Additional file 11. Table S4. Contingency table comparing mRNA-derived PCA-PAM50 subtypes to phosphoproteome clusters. The majority of the PCA-PAM50 subtype in each phosphoproteome cluster is highlighted in green. The Basal subtype in each of the Basal clusters is highlighted in red.

Additional file 12. Table S5. Differential biological pathways and functions between DTBC and LumA. (A) Genes and proteins that are significantly differentially expressed and share overlapping agreement in their upregulation (green) and downregulation (orange) between DTBC and LumA. (B) Genes that are significantly differentially expressed between DTBC and LumA and are linked to the IPA cell proliferation regulator network. The table concludes with the listing of 7 recognized IPA regulators, which are highlighted in yellow. (C) Proteins that are significantly differentially expressed between DTBC and LumA and are associated with the FOXC1 regulator network. (D) Predicted molecular functions by IPA for the differentially expressed phosphopeptides between DTBC and LumA.

## Data Availability

Data generated in this study, whole genome DNA sequencing, RNA sequencing, proteomics and phosphoproteomic data, will be submitted to dbGaP, the NCI Cancer Research Data Commons, and the ProteomeXChange. Further information and requests for analysis code, resources and reagents should be directed to and will be fulfilled by the lead contact, Hai Hu (H.Hu@wriwindber.org).

## Abbreviations

BC: Breast cancer
DTBC: Difficult-to-treat breast cancer
IHC: Immunohistochemical
PAM50: Prediction analysis of microarray 50 gene expression
Basal: Basal-like
Her2: Her2-enriched
LumB: Luminal B
LumA: Luminal A
ER: Estrogen receptor
PR: Progesterone receptor
HER2: Human epidermal growth factor receptor 2
TN: Triple-negative
LB1: Luminal B1
LB2: Luminal B2
LA: Luminal A
MS: Mass spectrometry
TCGA: The cancer genome atlas
IRB: Institutional review board
CBCP: Clinical breast care project
OCT: Optimal cutting temperature compound
PEN: Polyethylene-naphthalate
WGS: Whole genome sequencing
RNA-Seq: Total RNA sequencing
GDC: Genomic data commons
SNV: Single nucleotide variant
INDEL: Insertions/deletions
FDR: False discovery rate
SMG: Significantly mutated genes
DMG: Differentially mutated genes
TMB: Tumor mutational burden
SCNA: Somatic copy number alteration
TMT: Tandem mass tag
IMAC: Immobilized metal affinity chromatography
LC–MS/MS: Liquid chromatography with tandem mass spectrometry
QC: Quality control
PCA: Principal component analyses
OS: Overall survival
PFI: Progression-free interval
GSEA: Gene set enrichment analysis
MOGSA: Multi-omics gene set analysis
GSS: Gene-set scores
GLM: Generalized linear model
IPA: Ingenuity pathway analysis
CPTAC: Clinical proteomic tumor analysis consortium
FC: Fold change
