# Supplementary figures and images for: Proteogenomic characterization of difficult-to-treat breast cancer with tumor cells enriched through laser microdissection
Source: Breast Cancer Res. 2024 May 14;26:76. doi: 10.1186/s13058-024-01835-4 (PMC11094977; doi:10.1186/s13058-024-01835-4)

A

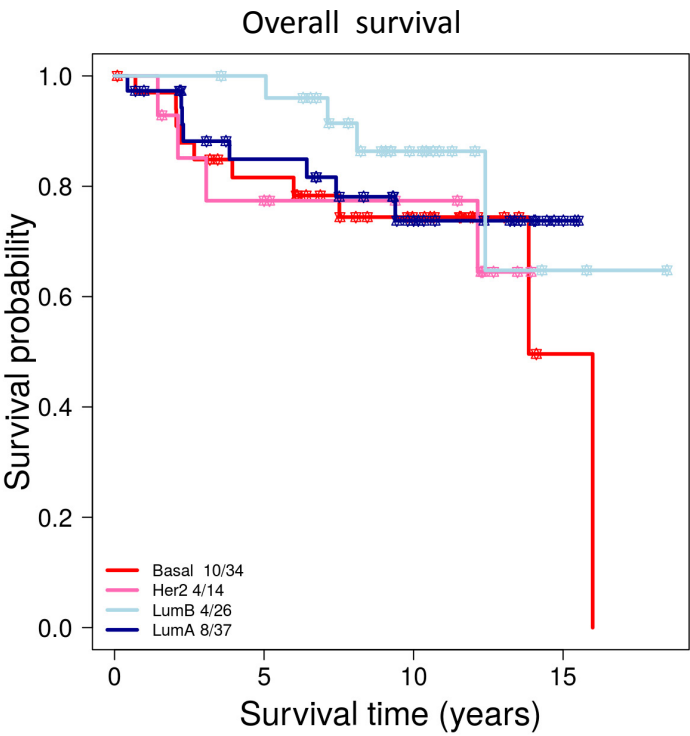

B

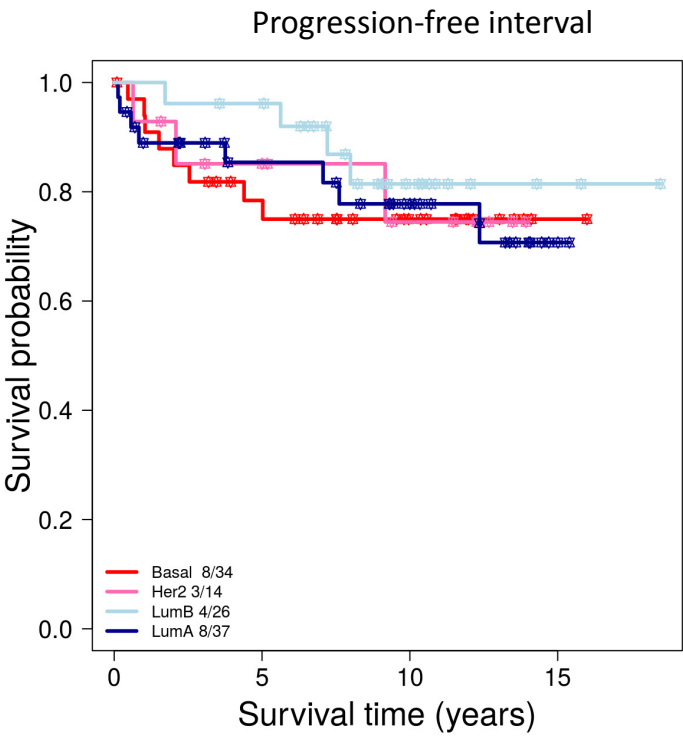

C

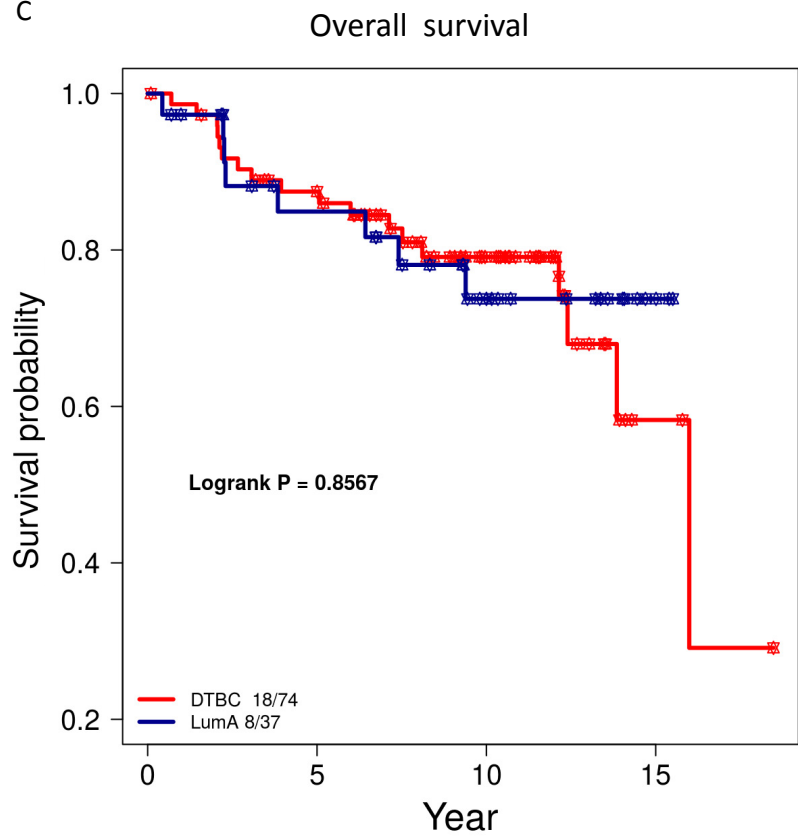

D

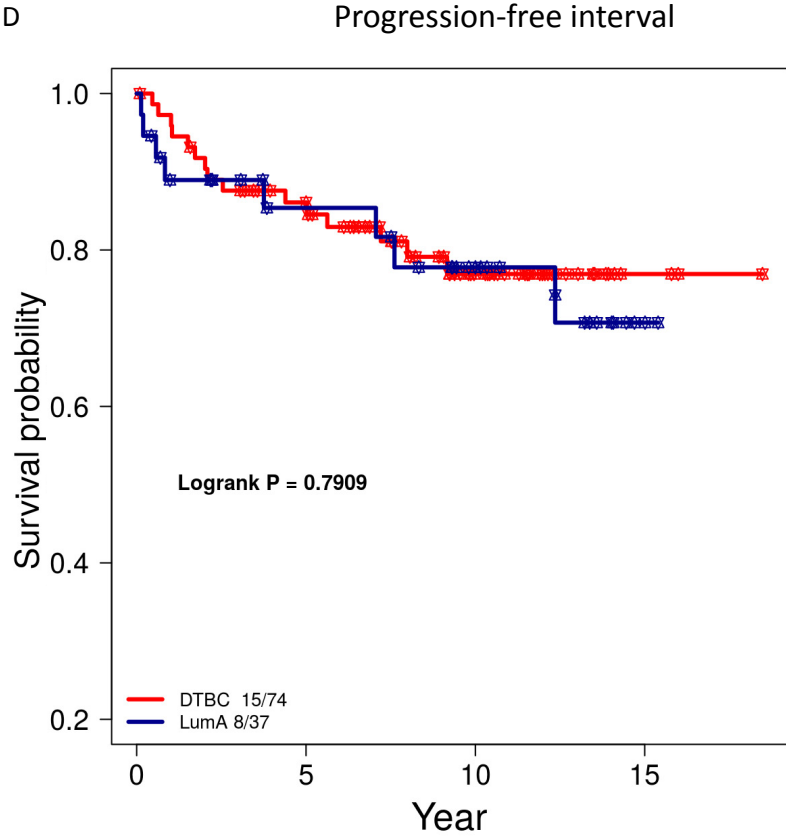

Supplement: Supplementary file 1 — Additional file 1. Figure S1. Kaplan–Meier Curves for the Cohort Based on PCA-PAM50 Subtypes and DTBC and LumA Subtype groups. Panels (A) and (B) display Kaplan–Meier curves for cumulative survival in years across PCA-PAM50 subtypes. Panels (C) and (D) present Kaplan–Meier curves for cumulative survival for DTBC and LumA subtypes. The endpoint of overall survival is used for panels (A) and (C) while the endpoint of progression-free interval is used for panels (B) and (D). The legends of the plots include the p-value and the count of events/total cases. [file 13058_2024_1835_MOESM1_ESM.pdf]

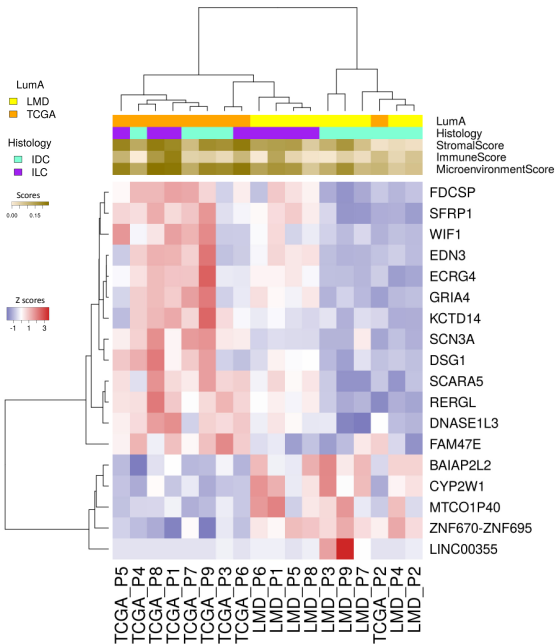

Supplement: Supplementary file 2 — Additional file 2. Figure S2. Unsupervised clustering of 9 LumA samples, a subset of the 34, using differentially expressed genes between LMD (yellow) and bulk processing (TCGA, orange). Histology, Stromal score, Immune score and Microenvironment score are provided as annotation. The corresponding pairs of LMD and TCGA samples were suffixed as P1, P2, etc [file 13058_2024_1835_MOESM2_ESM.pdf]

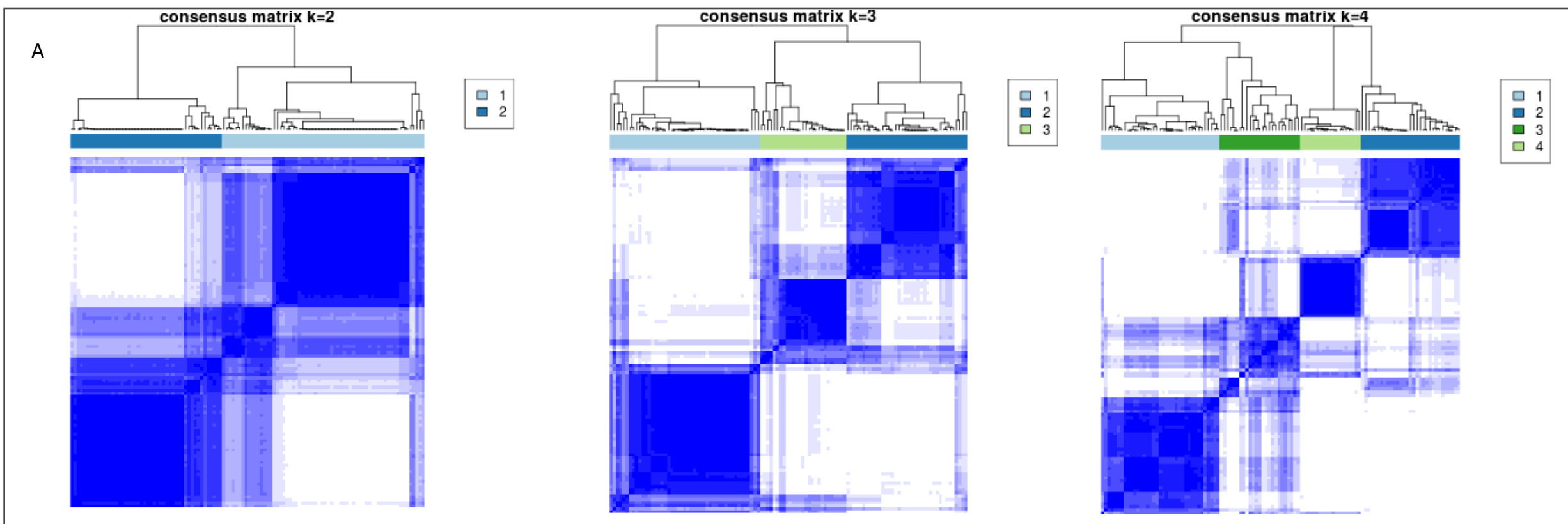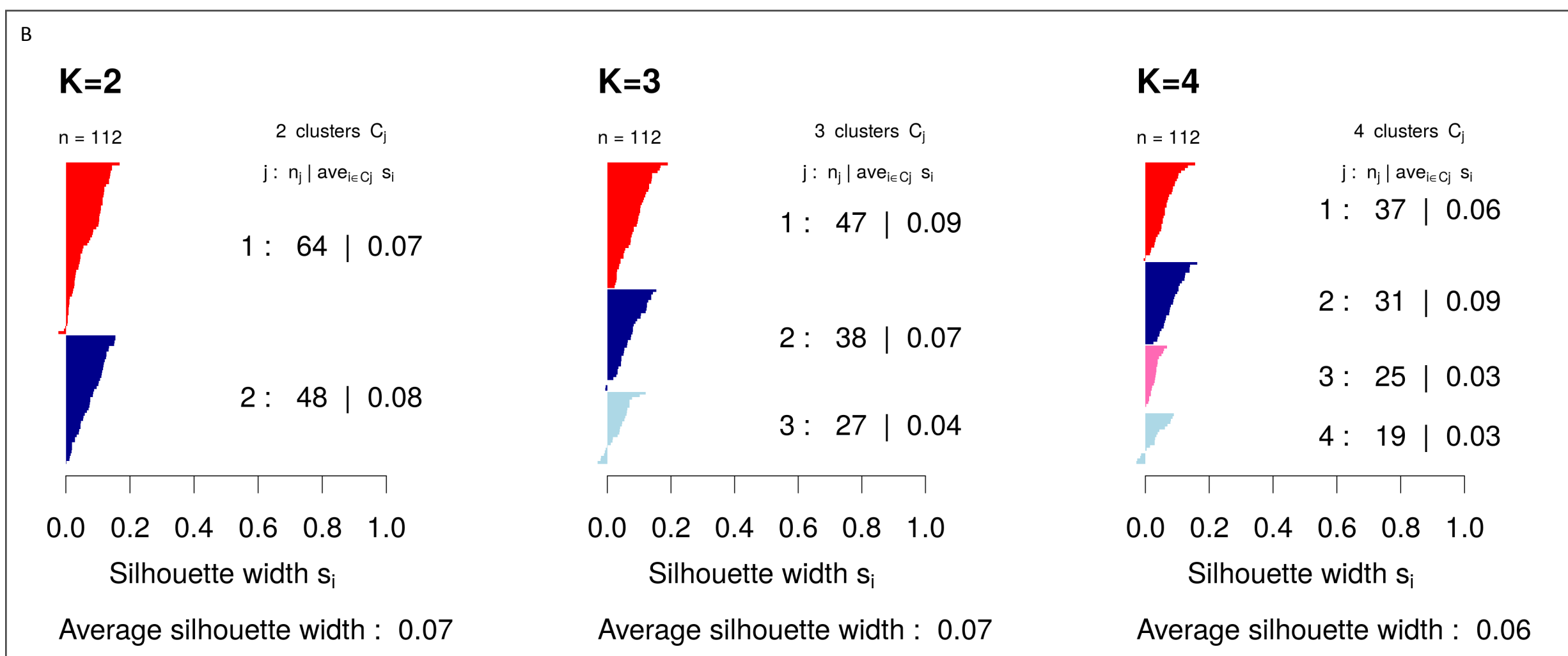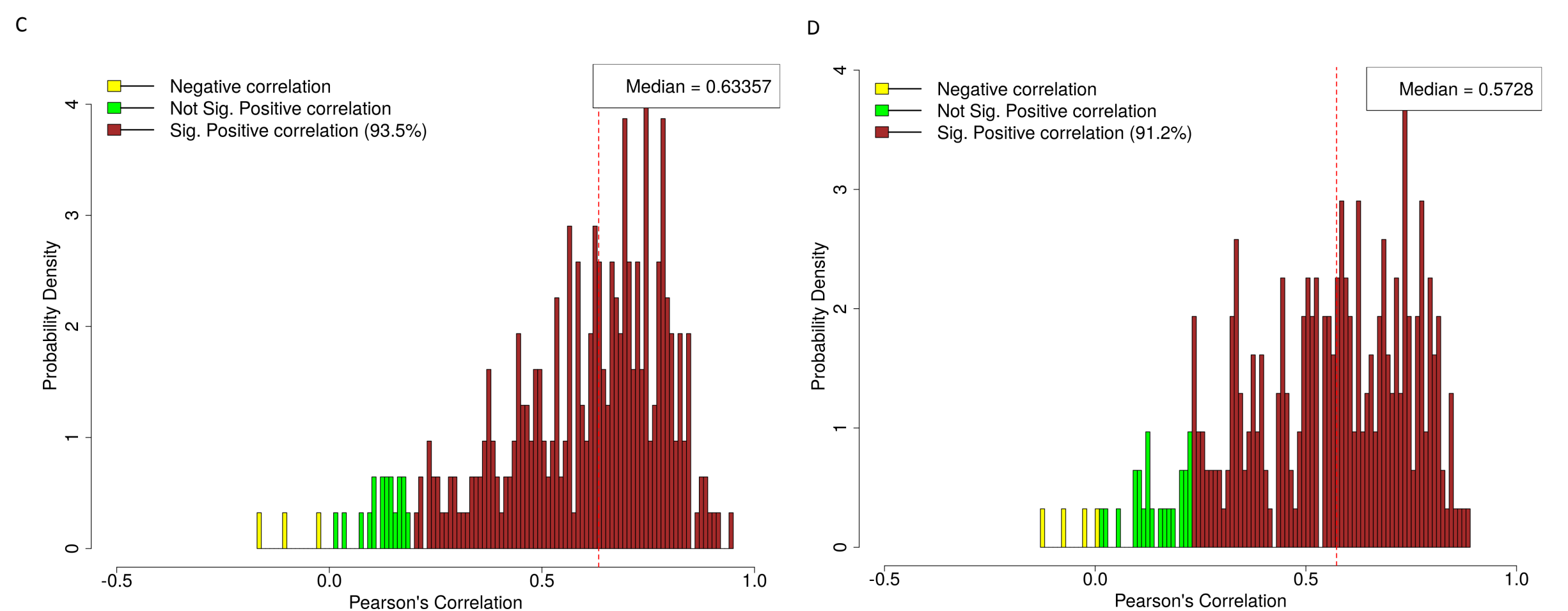

Supplement: Supplementary file 3 — Additional file 3. Figure S3. Quality metrics of K-means clustering and correlation analysis of the proteomics data. (A) Visualization of consensus matrices from K-means consensus clustering for K = 2, 3 and 4. (B) Silhouette plots are shown for K = 2, 3 and 4 clusters to evaluate the coherence of the clustering. K = 3 was selected as the optimal cluster because of its better separation and silhouette width. (C) The mRNA:protein correlations for 310 proteins overlapping between the LMD and CPTAC-2016 (D) datasets. [file 13058_2024_1835_MOESM3_ESM.pdf]

A

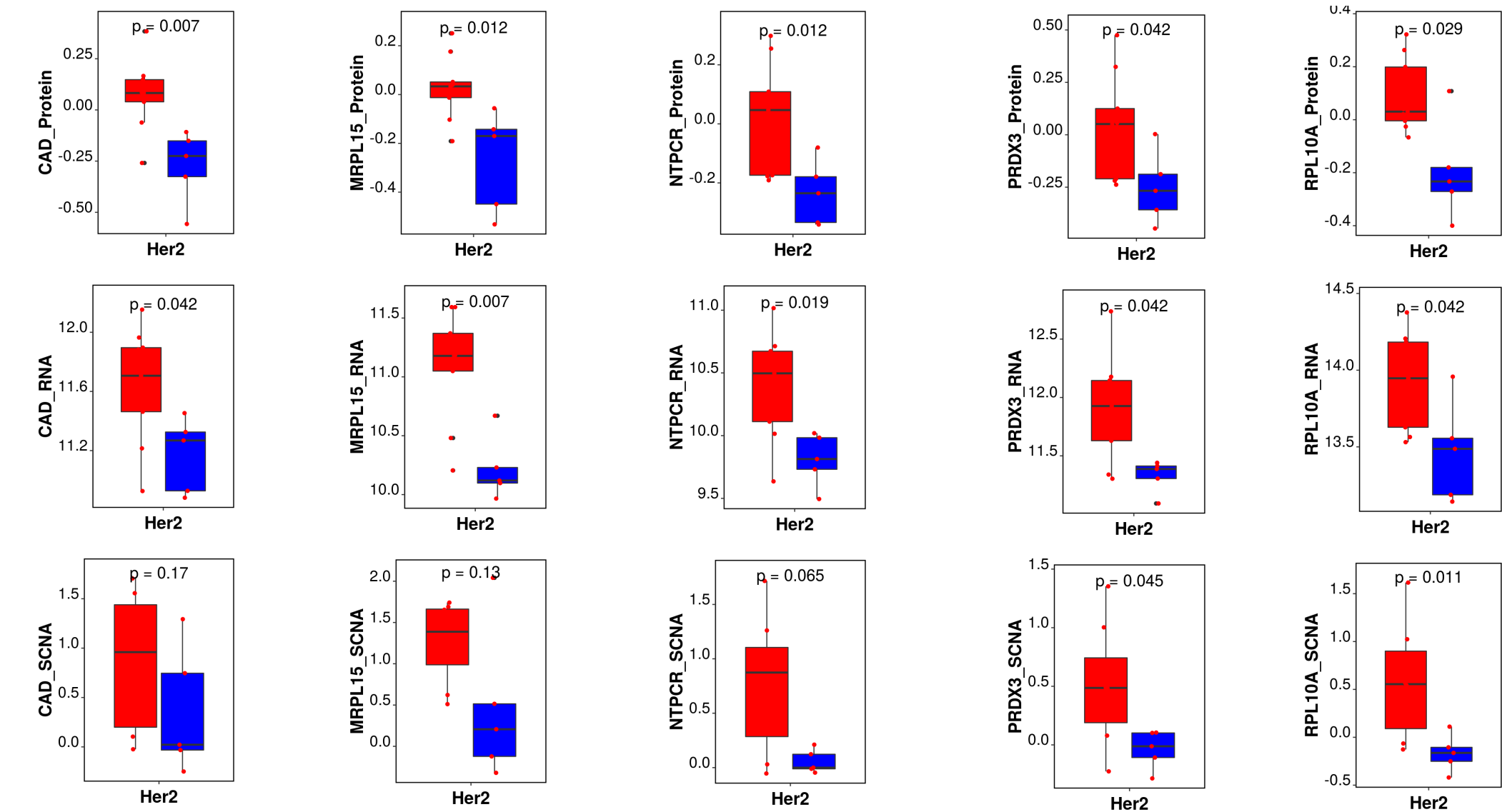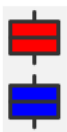

B

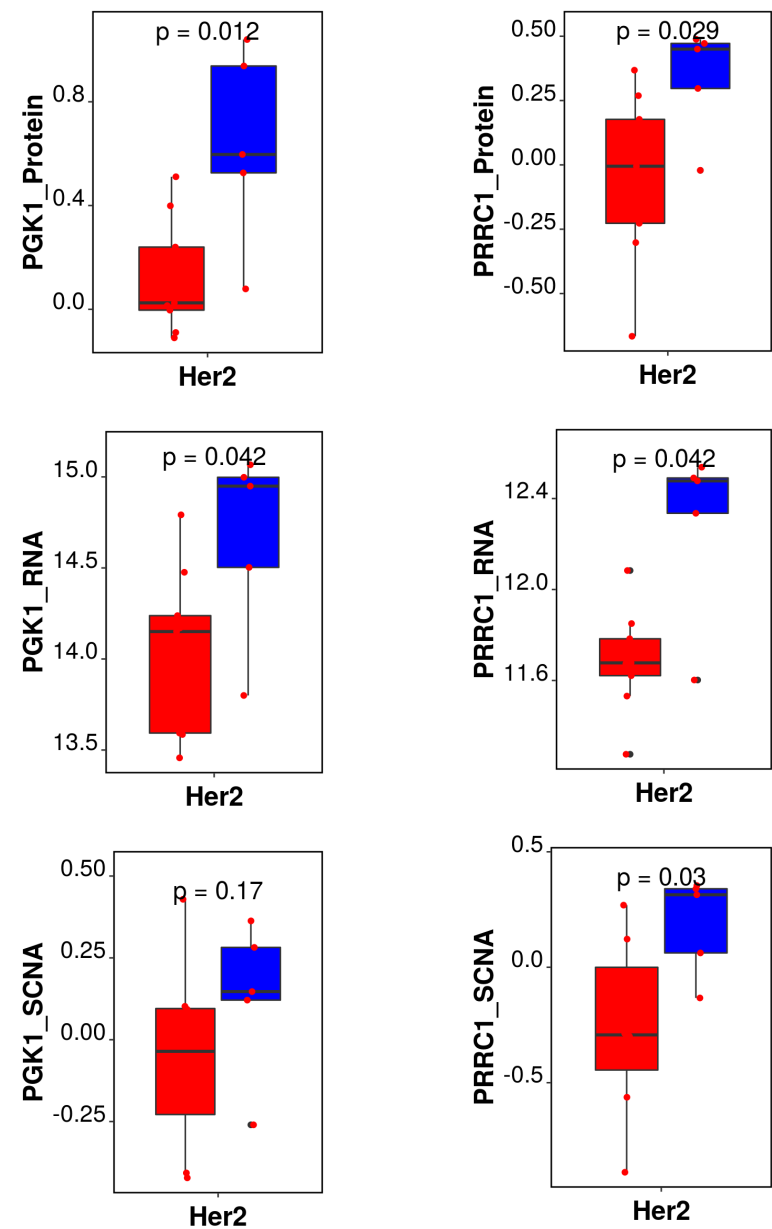

Supplement: Supplementary file 4 — Additional file 4. Figure S4. Multi-omics differences between Her2 cases of the Basal-enriched versus Luminal A-enriched protein clusters. Significantly up-regulated (A) and down-regulated proteins (B) in Her2 cases of the Basal-enriched versus Luminal A-enriched protein clusters, which also shows significant up and down-regulation, respectively, in the other two omics (transcriptomics (RNA) and genomics (SCNA)). Wilcoxon rank sum test p-value is given in the plot where p < 0.05 is considered significant. In the case of non-significant SCNA difference, the trended difference (p < 0.2) is included. [file 13058_2024_1835_MOESM4_ESM.pdf]

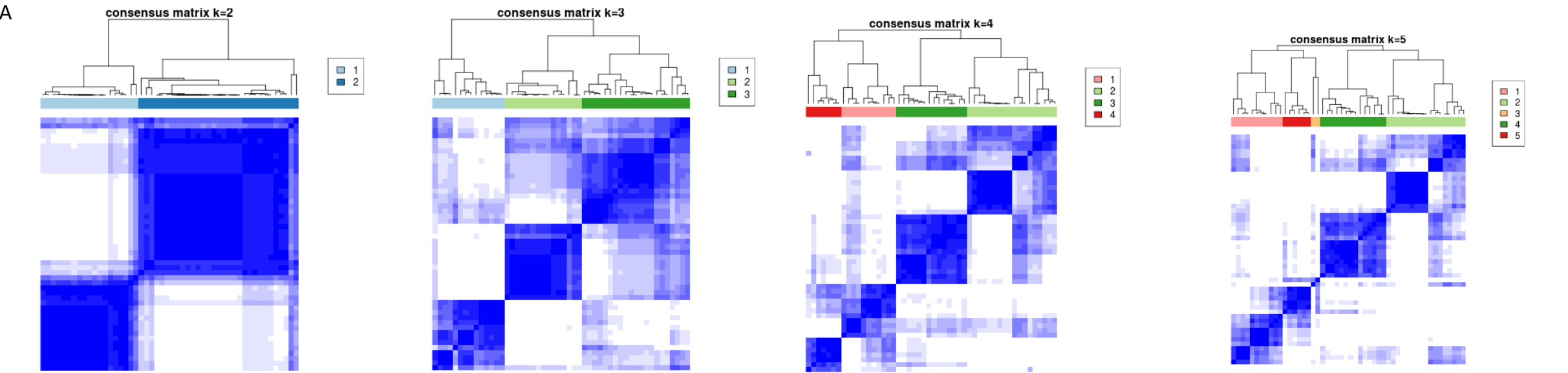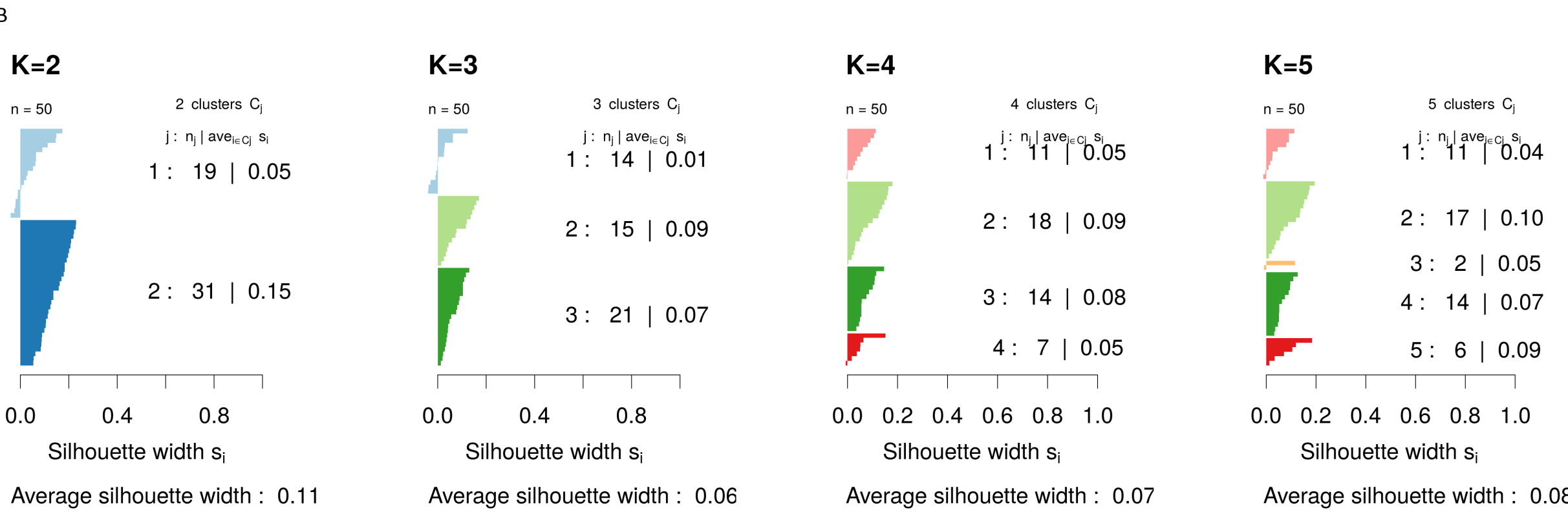

Supplement: Supplementary file 5 — Additional file 5. Figure S5. Quality metrics of K-means clustering of phosphoproteomics. (A) Visualization of consensus matrices from K-means consensus clustering for K = 2, 3, 4 and 5. (B) Silhouette plots are shown for K = 2, 3, 4 and 5 clusters to evaluate the coherence of the clustering. K = 4 was selected as the optimal cluster for its better separation and non-negative silhouette width. [file 13058_2024_1835_MOESM5_ESM.pdf]
