# Supplementary material for: Proteogenomic characterization of difficult-to-treat breast cancer with tumor cells enriched through laser microdissection
Source: Breast Cancer Res. 2024 May 14;26:76. doi: 10.1186/s13058-024-01835-4 (PMC11094977; doi:10.1186/s13058-024-01835-4)
Supplement: Supplementary file 7 — Additional file 7. Figure S7. Phosphoproteomic clusters with the names of Phosphopetides. This hierarchical clustering is identical to Fig. 4A but includes the names of all 245 phosphopeptides in the “GeneSymbol-ProteinEnsemblID-phosphosite” format. [file 13058_2024_1835_MOESM7_ESM.pdf]

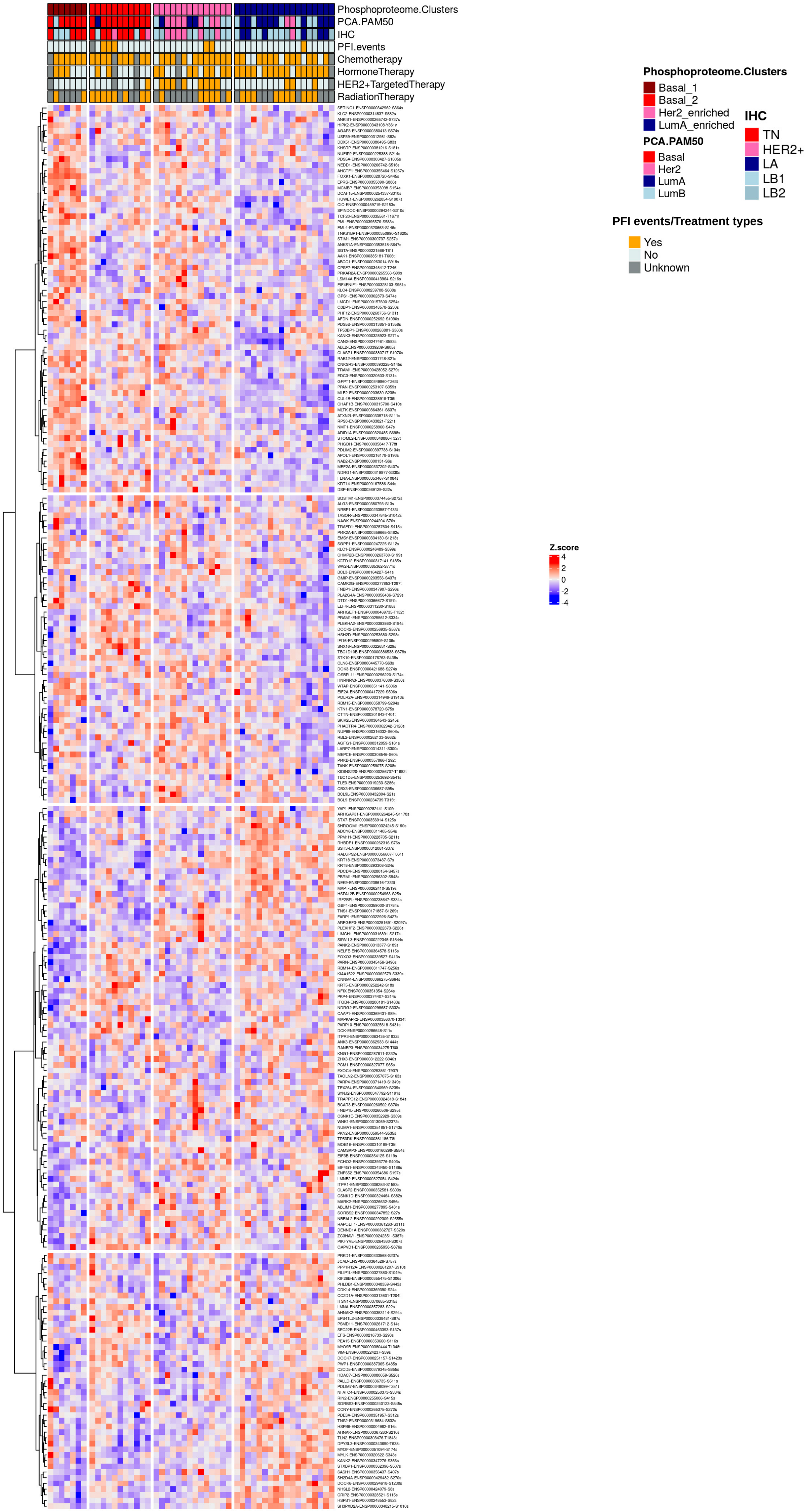
Phosphoproteome.Clusters
PCA.PAM50
IHC
PFI.events
Chemotherapy
HormoneTherapy
HER2+TargetedTherapy
RadiationTherapy
Phosphoproteome.Clusters
Basal_1
Basal_2
Her2_enriched
LumA_enriched
PCA.PAM50
Basal
Her2
LumA
LumB
IHC
TN
HER2+
LA
LB1
LB2
PFI events/Treatment types
Yes
No
Unknown
Z.score
4
2
0
-2
-4
