# Supplementary material for: Proteogenomic characterization of difficult-to-treat breast cancer with tumor cells enriched through laser microdissection
Source: Breast Cancer Res. 2024 May 14;26:76. doi: 10.1186/s13058-024-01835-4 (PMC11094977; doi:10.1186/s13058-024-01835-4)
Supplement: Supplementary file 10 — Additional file 10. Table S3. Contingency table comparing mRNA-derived PCA-PAM50 subtypes to proteome clusters. The majority of the PCA-PAM50 subtype in each proteome cluster is highlighted in green. The Her2 subtype separated in the Basal-enriched and LumA-enriched clusters is highlighted in red. [file 13058_2024_1835_MOESM10_ESM.docx]

**Supplementary Table S3. Contingency table comparing mRNA-derived PCA-PAM50 subtypes to proteome clusters.** The majority of the PCA-PAM50 subtype in each proteome cluster is highlighted in green. The Her2 subtype separated in the Basal-enriched and LumA-enriched clusters is highlighted in red.

|  | | Proteome clusters | | |  |
| --- | --- | --- | --- | --- | --- |
|  |  | Basal_enriched | LumB_enriched | LumA_enriched | Sum |
| PCA-PAM50 | Basal | 32 | 1 | 2 | 35 |
|  | Her2 | **9** | 1 | **5** | 15 |
|  | LumA | 2 | 7 | 27 | 36 |
|  | LumB | 4 | 18 | 4 | 26 |
|  | Sum | 47 | 27 | 38 | 112 |
