# Supplementary material for: Proteogenomic characterization of difficult-to-treat breast cancer with tumor cells enriched through laser microdissection
Source: Breast Cancer Res. 2024 May 14;26:76. doi: 10.1186/s13058-024-01835-4 (PMC11094977; doi:10.1186/s13058-024-01835-4)
Supplement: Supplementary file 11 — Additional file 11. Table S4. Contingency table comparing mRNA-derived PCA-PAM50 subtypes to phosphoproteome clusters. The majority of the PCA-PAM50 subtype in each phosphoproteome cluster is highlighted in green. The Basal subtype in each of the Basal clusters is highlighted in red. [file 13058_2024_1835_MOESM11_ESM.docx]

**Supplementary Table S4. Contingency table comparing mRNA-derived PCA-PAM50 subtypes to phosphoproteome clusters.** The majority of the PCA-PAM50 subtype in each phosphoproteome cluster is highlighted in green. The Basal subtype in each of the Basal clusters is highlighted in red.

|  | | Phosphoproteome clusters | | | |  |
| --- | --- | --- | --- | --- | --- | --- |
|  | | Basal_1 | Basal_2 | Her2_enriched | LumA_enriched | Sum |
| PCA-PAM50 | Basal | 6 | 10 | 0 | 0 | 16 |
|  | Her2 | 0 | 0 | 7 | 2 | 9 |
|  | LumA | 0 | 1 | 3 | 10 | 14 |
|  | LumB | 1 | 0 | 4 | 6 | 11 |
|  | Sum | 7 | 11 | 14 | 18 | 50 |
