# Supplementary material for: Proteogenomic characterization of difficult-to-treat breast cancer with tumor cells enriched through laser microdissection
Source: Breast Cancer Res. 2024 May 14;26:76. doi: 10.1186/s13058-024-01835-4 (PMC11094977; doi:10.1186/s13058-024-01835-4)
Supplement: Supplementary file 6 — Additional file 6. Figure S6. Kaplan–Meier curves of time to disease progression in years for the significantly (FC > 1.2 and FDR ≤ 0.2) differentially expressed phosphopeptides between 10 basal cases of the Basal_2 cluster (high relapse-risk) and 6 basal cases of the Basal_1 cluster(low relapse-risk). The high (> median) and low (< median) expression of all 40 up-regulated (A) and 36 down-regulated (B) phosphopeptides in the Basal_2 versus Basal_1 clusters with the end point of PFI. Gene name, P-value, phosphosite and the number of events/number of cases are given in each plot. [file 13058_2024_1835_MOESM6_ESM.pdf]

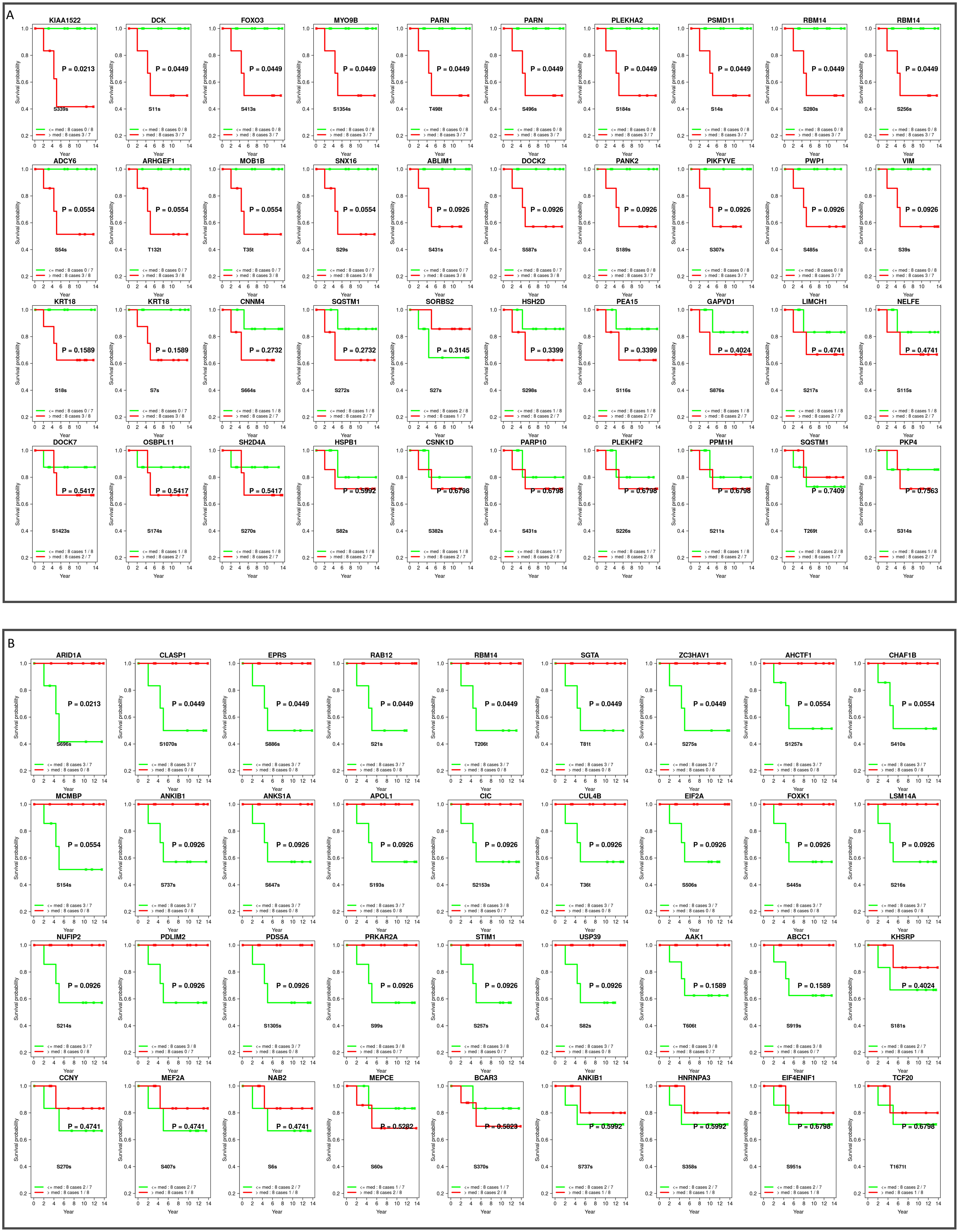
A
KIAA1522
P = 0.0213
S339s
DCK
P = 0.0449
S11s
FOXO3
P = 0.0449
S413s
MYO9B
P = 0.0449
S1354s
PARN
P = 0.0449
T498t
PARN
P = 0.0449
S496s
PLEKHA2
P = 0.0449
S184s
PSMD11
P = 0.0449
S14s
RBM14
P = 0.0449
S280s
RBM14
P = 0.0449
S256s
ADCY6
P = 0.0554
S54s
ARHGEF1
P = 0.0554
T132t
MOB1B
P = 0.0554
T35t
SNX16
P = 0.0554
S29s
ABLIM1
P = 0.0926
S431s
DOCK2
P = 0.0926
S587s
PANK2
P = 0.0926
S189s
PIKFYVE
P = 0.0926
S307s
PWP1
P = 0.0926
S485s
VIM
P = 0.0926
S39s
KRT18
P = 0.1589
S18s
KRT18
P = 0.1589
S7s
CNNM4
P = 0.2732
S664s
SQSTM1
P = 0.2732
S272s
SORBS2
P = 0.3145
S27s
HSH2D
P = 0.3399
S298s
PEA15
P = 0.3399
S116s
GAPVD1
P = 0.4024
S876s
LIMCH1
P = 0.4741
S217s
NELFE
P = 0.4741
S115s
DOCK7
P = 0.5417
S1423s
OSBPL11
P = 0.5417
S174s
SH2D4A
P = 0.5417
S270s
HSPB1
P = 0.5992
S82s
CSNK1D
P = 0.6798
S382s
PARP10
P = 0.6798
S431s
PLEKHF2
P = 0.6798
S226s
PPM1H
P = 0.6798
S211s
SQSTM1
P = 0.7409
T269t
PKP4
P = 0.7563
S314s
B
ARID1A
P = 0.0213
S696s
CLASP1
P = 0.0449
S1070s
EPRS
P = 0.0449
S886s
RAB12
P = 0.0449
S21s
RBM14
P = 0.0449
T206t
SGTA
P = 0.0449
T81t
ZC3HAV1
P = 0.0449
S275s
AHCTF1
P = 0.0554
S1257s
CHAF1B
P = 0.0554
S410s
MCMBP
P = 0.0554
S154s
ANKIB1
P = 0.0926
S737s
ANKS1A
P = 0.0926
S647s
APOL1
P = 0.0926
S193s
CIC
P = 0.0926
S2153s
CUL4B
P = 0.0926
T36t
EIF2A
P = 0.0926
S506s
FOXK1
P = 0.0926
S445s
LSM14A
P = 0.0926
S216s
NUFIP2
P = 0.0926
S214s
PDLIM2
P = 0.0926
S737s
PDS5A
P = 0.0926
S1305s
PRKAR2A
P = 0.0926
S99s
STIM1
P = 0.0926
S257s
USP39
P = 0.0926
S82s
AAK1
P = 0.1589
T606t
ABCC1
P = 0.1589
S919s
KHSRP
P = 0.4024
S181s
CCNY
P = 0.4741
S270s
MEF2A
P = 0.4741
S407s
NAB2
P = 0.4741
S6s
MEPCE
P = 0.5282
S60s
BCAR3
P = 0.5823
S370s
ANKIB1
P = 0.5992
S737s
HNRNPA3
P = 0.5992
S358s
EIF4ENIF1
P = 0.6798
S951s
TCF20
P = 0.6798
T1671t
Survival probability
Year
